# Synthesis and molecular docking studies of quinoline derivatives as HIV non-nucleoside reverse transcriptase inhibitors

**DOI:** 10.3906/kim-2004-14

**Published:** 2020-12-16

**Authors:** Nivedita BHARDWAJ, Diksha CHOUDHARY, Akashdeep PATHANIA, Somesh BARANWAL, Pradeep KUMAR

**Affiliations:** 1 Department of Pharmaceutical Sciences and Natural Products, Central University of Punjab, Bathinda India; 2 Department of Microbiology, Central University of Punjab, Bathinda India; 3 Department of Pharmaceutical Engineering and Technology, Indian Institute of Technology (Banaras Hindu University), Varanasi India

**Keywords:** Quinoline, HIV, NNRTIs, pyrimidine, pyrazoline

## Abstract

Quinoline moiety is an important scaffold in the field of drug discovery and drug development, with a wide range of pharmacological activities. Quinoline derivatives are potent inhibitors for reverse transcriptase, which is responsible for the conversion of single-stranded viral RNA into double-stranded viral DNA.In the present study, we have designed and synthesized 2 series, namely pyrazoline and pyrimidine containing quinoline derivatives as non nucleoside reverse transcriptase inhibitors (NNRTIs). Eleven compounds were synthesized and characterized by 1H and 13C NMR and mass spectrophotometry. The synthesized compounds were also docked on an HIV reverse transcriptase binding site (PDB: 4I2P); most of these compounds showed good binding interactions with the active domain of the receptor. Most of the compounds displayed a docking score higher than those of standard drugs. Among the synthesized quinoline derivatives, compound **4** exhibited the highest docking score (–10.675).

## 1. Introduction

It has been over three decades since HIV, the causative agent for acquired immunodeficiency syndrome (AIDS), was identified. From the beginning of the global pandemic of HIV in the early 1980s, an estimated 78 million people have been infected with HIV; about 39 million people have died of AIDS-related causes [1]. There are 2 major types of HIV, HIV-1 and HIV-2, out of which HIV-1 is the more virulent and pathogenic.

HIV reverse transcriptase enzyme specifically synthesizes double-stranded DNA from single-stranded viral RNA and is an important target for anti-HIV drug development. In the early 1990s, potent HIV reverse transcriptase inhibitors (RTIs) with significant clinical activity were developed. Nonnucleoside reverse transcriptase inhibitors (NNRTIs) are leading drugs in the treatment of HIV-1 infections [2]. NNRTIs are capable of reducing the viral reservoirs in the brain due to their high lipophilicity because of the absence of sugar molecules in their structure. Currently, highly active antiretroviral therapy (HAART) combination therapy is used for the treatment of HIV to avoid the development of resistance against a single drug, but it also causes psychiatric and neurological side effects [3]. Hence, there is a dire need for novel anti-HIV agents with fewer side effects.

Drug discovery is a very long and costly procedure. It requires 10–15 years and billions of dollars to discover a new drug. CADD tools significantly reduce time and monetary investment in drug discovery. Molecular docking is one of the most important tools of CADD used in drug discovery today to understand drug-receptor interactions, the binding affinity of drugs, and orientation of drug molecules to the target site. This helps in better prediction of activity and reduction in side effects, and is a rational approach to drug design [4].

Quinoline and pyrimidine are important scaffolds for anti-HIV drugs. Hameed et al. designed and synthesized quinoline-based chalcones as HIV reverse transcriptase (RT) inhibitors. Molecular docking studies were performed for the synthesized compounds to determine their binding affinity in the active site of the enzyme. HIV-RT bioassay was used to access the biological activity of the synthesized compounds and to verify the results of molecular docking studies, which indicated that chloro- and bromo-substituted quinoline-containing compounds showed potent cytotoxicity against HIV-RT [5]. Anti-HIV drug-like elvitegravir is a quinoline derivative, whereas efavirenz contains a benzoxazine (quinoline-like) scaffold. Rilpivirine, etravirine, and dapivirine possess a pyrimidine scaffold [6]. Pyrazole is also an important scaffold of anti-HIV drugs. The pyrazole ring as the core structure enhances the solubility and bioavailability of anti-HIV agents [7,8]. Quinoline and pyrimidine derivatives reported as NNRTIs are summarized in Tables 1 and 2, respectively [9–18].

**Table 1:** Quinoline derivatives as NNRTIs.

S. No.	Compounds	IC50 (µM)	Reference
1.	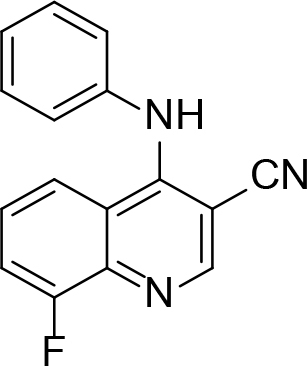	<8.00	[8]
2.	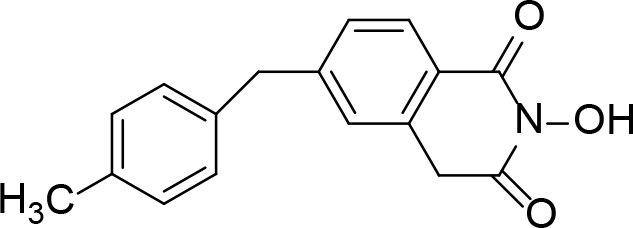	1.20	[9]
3.	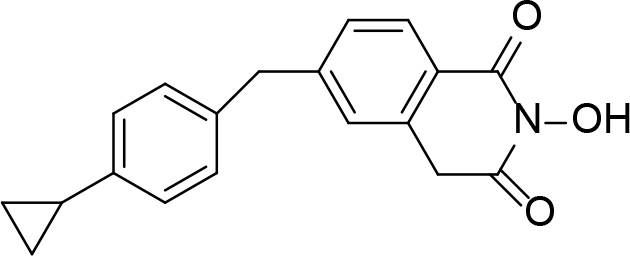	0.60	
4.	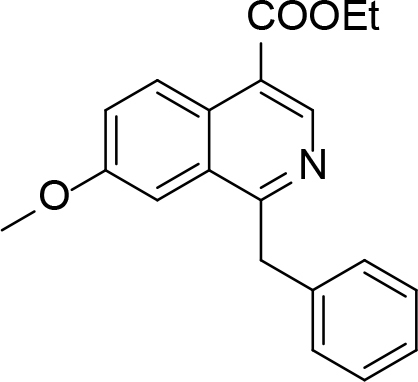	~10.00	[10]
5.	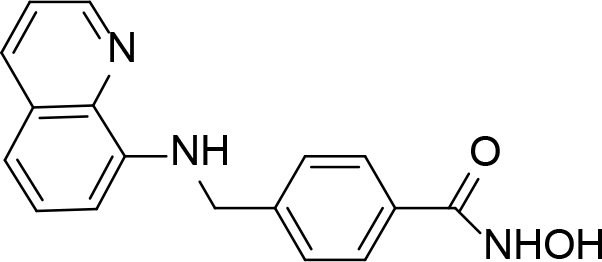	0.29	[11]

**Table 2:** Pyrimidine derivatives as NNRTIs.

S. No.	Compound	IC50 (µM)	Reference
1	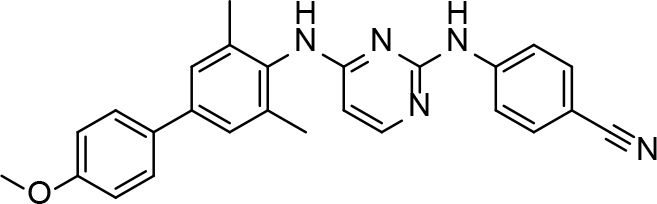	0.03	[12]
2	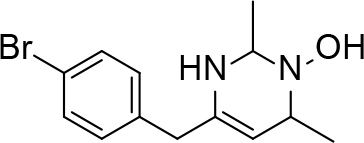	0.01	[13]
3	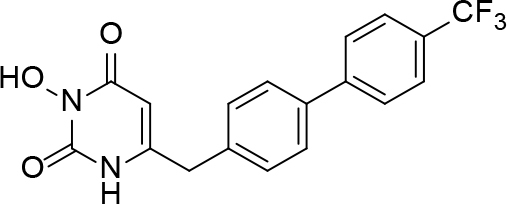	0.05	[14]
4.	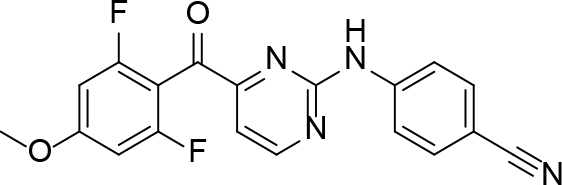	0.04	[15]
5.	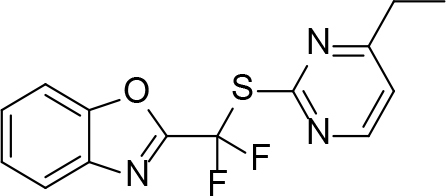	6.40	[7]
6.	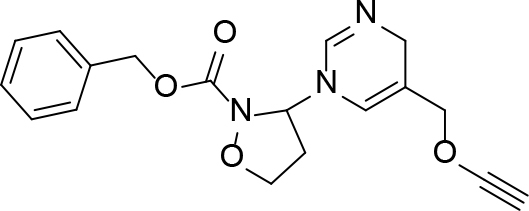	0.10	[16]
7.	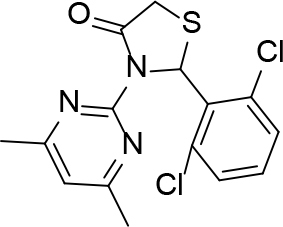	10.00	[17]

In light of the abovementioned facts, quinoline derivatives were synthesized in the present study and then docked in the active site of HIV reverse transcriptase protein to explore their binding affinity and their interactions with the key amino acid residues of the active site of HIV reverse transcriptase.

## 2. Materials and methods

All of the reagents were purchased from commercial sources and were used without any additional purification. Precoated Merck TLC plates were utilized for monitoring the progress of the reaction (Merck & Co., Inc., Kenilworth, NJ, USA). Melting points were measured with a Stuart melting point apparatus (SMP-11) with an open glass capillary tube and are uncorrected (Cole-Parmer Ltd., Stone, UK). Infrared (IR) spectra of compounds were recorded on a Bruker FT-IR spectrophotometer (Bruker BioSpin Corp., Billerica, MA, USA). Mass spectra were recorded on a Shimadzu GCMS-QP2010 with EI mode (Shimadzu Corp., Kyoto, Japan). 1H NMR (proton nuclear magnetic resonance) and 13C NMR (carbon nuclear magnetic resonance) spectra were recorded with the help of a Brucker Avance II (400 MHz) NMR spectrometer using CDCl3 as a solvent (Bruker BioSpin Corp.). TMS (tetramethylsilane) was used as an internal reference standard (δ = 0). IR, NMR and mass spectra of the synthesized compounds are provided as supplementary information.

### 2.1. Synthesis of compounds

#### 2.2.1. General procedure for the synthesis of 2-chloroquinoline-3-carbaldehyde (1)

POCl3 (6.9 mL,74.07 mM) was added by drops via a dropping funnel to DMF (1.09 mL, 14.81 mM) at 0–5 °C; the mixture was stirred for 5 min. Acetanilide (1 g, 7.40 mM) was then added to the reaction mixture and the resulting solution was refluxed for 9 h at 90 °C. The reaction mixture was cooled to room temperature and then poured into crushed ice and stirred. Precipitate appeared at once, and was filtered, washed with water, and dried. The crude compound was then purified by column chromatography [19].

Pale yellow solid (yield 92%), m.p.: 142–144°C, IR (KBr) cm–1: C=N stretch (2359), Aldehyde C=O stretch (1688), C-Cl stretch (759), C-CN stretch (1044), C=C stretch (1615), mass (EI): calculated for C10H6ClNO: 191.01 [M]+, found at 191,1H NMR (CDCl3, 400 MHz, δ with TMS = 0): 7.91(1H, t, J = 8 Hz), 7.91(1H, t, J = 12 Hz), 7.98 (1H, d, J = 8 Hz), 8.09 (1H, d, J = 8 Hz ),8.76 (1H, s),10.57 (1H, s),13CNMR (CDCl3, 100 MHz) δ (ppm): 189.17, 150.12, 149.62, 133.61, 129.73, 128.64, 128.15, 126.56, 126.41.

#### 2.2.2. General procedure for the synthesis of chalcones (2 and 3)

2-chloroquinoline-3-carbaldehyde (1 mM) was added to substituted acetophenones (1 mM). After adding 5mL ethanol, the mixture was stirred until the reactants were completely dissolved. Then, 20% NaOH (1mL) was added by drops to it. The reaction mixture was stirred overnight and the precipitate that appeared was filtered, washed with water, dried, and recrystallized using ethanol and DCM. The reaction progress was monitored by TLC using petroleum ether:ethyl acetate (7:3) [20].

**1-(4-bromophenyl)-3-(2-chloroquinolin-3-yl)-1- prop-en-1-one (2)**

Yellow precipitate (yield = 84%), m.p.: 172–174°C, mass (EI): calculated for C18H11BrClNO: 372 gm/mol [M+], found at 372.

**3-(2-chloroquinolin-3-yl)-1-(p-tolyl)prop-2-en-1-one(3a)**

Orange precipitate (yield: 87%) m.p.: 153–155°C, mass (EI): calculated for C19H14ClNO: 307.08 gm/mol [M+], found at 307.

#### 2.2.3. General procedure for the synthesis of pyrimidine derivatives (4–8, series I)

A mixture of chalcone (2a or 2b) (0.50 mM), substituted guanidine hydrochloride (0.80 mM) and sodium carbonate (1.34 mM) was dissolved in DMF (3 mL). The reaction mixture was refluxed overnight at 90 °C with stirring. The reaction progress was monitored by TLC using pet ether:ethyl acetate (7:3) as a mobile phase. After the completion of the reaction, the mixture was cooled and extracted with water and ethylacetate. The product obtained was evaporated and purified by column chromatography and recrystallized by DCM and ethanol [21].

**4-(4-bromophenyl)-6-(2-chloroquinoline-3-yl)pyrimidin-2amine (4)**

Greenish yellow solid (yield: 87%), m.p.: 210–212 °C, IR (KBr)cm–1: N-H stretch (3500), C-Cl stretch (772), C-Br (685), C-CN stretch (1084), C=C stretch (1645), mass (EI): calculated for C19H12BrClN4: 411.99 [M+], found at 412, 1H NMR (CDCl3, 400MHz, δ (ppm) with TMS = 0): 8.50 (s, 1H), 8.12 (s, 1H), 7.99 (d, J = 8 Hz, 1H), 7.95 (d, J = 8 Hz, 1H), 7.91–7.86 (m, 1H), 7.84–7.82 (m, 1H),7.74 (d, J = 8 Hz, 2H), 7.65 (d, J = 8 Hz, 2H), 5.28 (s, 2H), 13 CNMR (CDCl3, 100 MHz) δ with TMS = 0: 164.52, 164.44 , 163.53, 149.54, 148.85, 136.81,135.06, 132.13,131.20, 130.34, 129.55, 129.43, 129.27, 127.76, 127.66, 124.80, 102.67.

**4-(4-bromophenyl)-6-(2-chloroquinoline-3-yl)-N-methylpyrimidin-2amine(5)**

Greenish blue solid (yield: 59%) m.p.: 207–209 °C, mass (EI): calculated for C20H14BrClN4: 424 gm/mol [M+], found fragmented peak at 412, 1H NMR (CDCl3, 400 MHz, δ with TMS = 0): 8.17 (s, 1H), 7.99 (s, 1H), 7.87 (d, J = 8Hz, 1H), 7.81 (d, J = 8 Hz, 1H), 7.74 (m, 1H), 7.63 (m, 1H), 7.58 (d, J = 8 Hz, 2H), 7.55 (d, J = 12 Hz, 2H), 4.60 (s, 1H), 3.64–3.55 (m, 3H).

**4-(4-bromophenyl)-6-(2-chloroquinoline-3-yl)-N-phenylpyrimidin-2-amine(6)**

White solid (yield 68%), m.p.: 222–225 °C, mass (EI): calculated for C25H15BrClN3: 471 gm/mol [M+], found peak at 473, 1H NMR (CDCl3, 400 MHz, δ with TMS = 0): 8.61 (d, J = 4 Hz, 2H), 8.12 (s, 1H), 8.10 (s, 1H) , 8.05 (d, J = 8Hz, 1H), 8.03 (d, J = 8 Hz, 1H), 7.92–7.78 (m, 1H), 7.77–7.63 (m, 1H), 7.99 (d, J = 10 Hz, 2H), 7.47 (d, J = 4 Hz, 2H), 7.32–7.26 (m, 3H).

**4-(2-chloroquinolin-3-yl)-6-(p-tolyl)pyrimidin-2-amine(7)**

Greenish yellow solid (yield 47%), m.p.: 203–205 °C, mass (EI): calculated for C20H15ClN4: 346 gm/mol [M+] peak, found fragmented peak at 316, 1H NMR (CDCl3, 400MHz, δ with TMS = 0): 8.46 (s,1H), 8.17 (s, 1H), 8.15 (d, J = 8 Hz, 1H), 7.97 (d, J = 8 Hz, 1H), 7.93–7.87 (m, 1H), 7.83–7.79 (m,1H), 7.67 (d, J = 6 Hz, 2H), 7.61 (d, J = 6 Hz, 2H), 7.54 (s, 2H), 2.07 (s, 3H).

**2-chloro-3-(2-phenyl-6-(p-tolyl)pyrimidin-4-yl)quinoline(8)**

White solid (yield: 45%) m.p.: –200–203 °C, mass (EI): calculated for C26H18ClN3: 407.90 gm/mol [M+].Found peak at 407, 1H NMR (CDCl3, 400 MHz, δ with TMS=0): 8.93 (s, 1H), 8.61 (d, J=10Hz, 2H), 8.59 (s,1H), 8.41 (d, J=6Hz,1H), 8.33 (d, J=12Hz,1H), 8.19–8.08 (m,1H), 7.96–7.93 (m,1H), 7.78–7.75 (m,3H), 7.59 (d, J=10Hz, 2H), 7.53 (d, J=8Hz, 2H), 3.29 (s,3H).

#### 2.2.4. General procedure for the synthesis of pyrazoline derivatives (series 2):

Chalcone (0.50 mM) was added to substituted phenylhydrazine hydrochloride (0.80 mM). After adding 5 mL methanol, the mixture was stirred until the reactants were completely dissolved. The reaction was stirred on a magnetic stirrer for 4–5 h at 50–60 °C. Reaction progress was monitored by TLC using pet ether:ethyl acetate (7:3). After 4–5 h, precipitate appeared, which was filtered and washed with water. The filtered precipitate was dried and purified by column chromatography and recrystallized by methanol and DCM [22]. The synthesized compounds are shown in Table 3.

**Table 3:** Synthesizedquinoline derivatives.

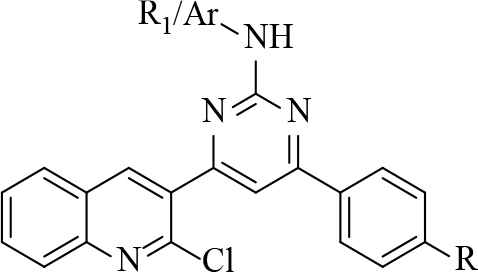			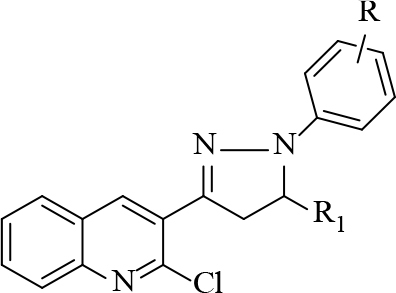		
Series 1			Series 2		
Compound	R	R1/Ar	Compound	R	R1
4	Br	H	9	3-Cl	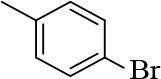
5	Br	CH3	10	3-F	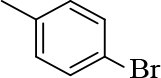
6	Br	C6H5	11	4-Br	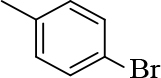
7	CH3	H	12	4-F	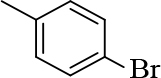
8	CH3	C6H5	13	3-Br	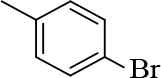
			14	4-Br	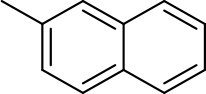

**3-(5-(4-bromophenyl)-1-(3-chlorophenyl)-4,5-dihydro-1H-pyrazol-3-yl)-chloroquinoline (9)**

Light green (yield: 65%), m.p.: 218–220 °C,1H NMR [CDCl3, 400 MHz, δ (ppm) with TMS = 0]: 3.14 (1H, dd, *J* *12* *=* 4 Hz *, J* *13* *=* 16 Hz), 4.07 (1H, dd, *J* *12* *=* 4 Hz *, J* *13* *=* 16 Hz), 5.72 (1H, dd, *J* *12* *=* 8 Hz *, J* *13* *=* 12 Hz), 6.68 (1H, dd, *J =* 4 Hz *)* , 6.80 (1H, dd, *J =* 4 Hz), 7.06 (1H, t, *J =* 8 Hz), 7.24 (1H, t, *J =* 4 Hz), 7.53 (1H, s), 7.51 (2H, m), 7.60 (2H, m), 7.71 (2H, m), 7.88 (1H, s), 8.03 (1H, d, *J =* 12 Hz),13C NMR (100 MHz, CDCl3) δ (ppm) with TMS=0: 158.6, 135.8, 132.0, 131.9, 131.8, 130.9, 130.32, 128.25, 127.98, 127.86, 127.65, 127.49, 119.84, 113.64, 111.02, 108.21, 107.4, 61.25, 42.3, 32.0, 29.7, 29.7, 14.2, mass (EI): calculated for C24H16BrCl2N3: 494.99 gm/mol [M+]; found at 495, [M+2] peak at 497.

**3-(5-(4-bromophenyl)-1-(3-fluorophenyl)-4,5-dihydro-1H-pyrazol-3-yl)-2-chloroquinoline (10)**

Yellowish green (yield: 57.14%), m.p.: 215–218 °C, 1H NMR [CDCl3, 400 MHz, δ (ppm) with TMS = 0]: 3.14 (1H, dd, *J* *12* *=* 4 Hz *, J* *13* *=* 16 Hz), 4.07 (1H, dd, *J* *12* *=* 12 Hz, *J* *13* *=* 20 Hz), 5.70 (1H, m), 6.54 (1H, m), 6.68 (1H, s), 7.12 (1H, m), 7.51 (1H, m), 7.52 (2H, d, *J =* 4 Hz), 7.58 (1H, s), 7.60 (2H, d, *J =* 8 Hz), 7.69 (1H, d, *J =* 8 Hz), 7.72 (1H, d, *J =* 4 Hz), 7.92 (1H, s), 8.02 (1H, d, *J =* 8 Hz),13C NMR (100 MHz, CDCl3) δ (ppm) with TMS = 0: 165.06, 162.64, 148.56, 147.32, 147.22, 136.01, 135.91, 131.95, 130.93, 127.97, 127.64, 127.47, 108.61, 108.60, 106.46, 101.11, 100.84, 77.42, 77.10, 76.78, 61.42, 58.97, 42.29, 42.23, mass (EI): calculated for C24H16BrClFN3: 479.02 gm/mol [M+]; found at 479, [M+2] peak at 481.

**3-(1-(3-bromophenyl)-5-(4-bromophenyl)-4,5-dihydro-1H-pyrazol-3-yl)-2-chloroquinoline (11)**

Light green (yield: 50%), m.p.: 212–215 °C,1H NMR [CDCl3, 400 MHz, δ (ppm) with TMS = 0]; 3.07 (1H, dd, *J* = 4 Hz), 3.98 (1H, t, *J* *23* = 12 Hz), 5.64 (1H, dd, *J* = 4 Hz), 6.61 (1H, d, *J =* 8 Hz), 6.84 (1H, d, *J* = 8 Hz), 6.93 (1H, t, *J* = 8 Hz), 7.31 (1H, s), 7.41 (3H, t, *J* = 4 Hz), 7.50 (2H, d, *J* = 4 Hz), 7.63 (2H, d, *J* = 4 Hz), 7.80 (1H, s), 7.92 (1H,d, *J* = 8 Hz), mass (EI): calculated for C24H16Br2ClN3: 538.94 gm/mol [M]+; found 539, [M+2] peak at 541.

**3-(5-(4-bromophenyl)-1-(4-fluorophenyl)-4,5-dihydro-1H-pyrazol-3-yl)-2-chloroquinoline (12)**

Green (yield: 70%), m.p.: 216–221 °C,1H NMR: [CDCl3, 400 MHz, δ (ppm) with TMS = 0] 3.12 (1H, dd, *J =* 8 Hz), 4.06 (1H, t, *J =* 12 Hz), 5.67 (1H, t, *J =* 8 Hz), 6.94 (4H, dd, *J =* 4 Hz), 7.50 (3H, d, *J =* 8 Hz), 7.58 (2H, d, *J =* 8 Hz), 7.72 (2H, m), 7.95 (1H, s), 8.05 (1H, d), mass (EI): calculated for C24H16BrClFN3: 479.02 gm/mol [M+]; found at 479, [M+2] peak at 481.

**3-(1,5-bis(4-bromophenyl)-4,5-dihydro-1H-pyrazol-3-yl)-2-chloroquinoline (13)**

Dark green (yield: 60%), m.p.: 219–222 °C, 1H NMR [CDCl3, 400 MHz, δ (ppm) with TMS = 0]: 3.15 (1H, dd, *J =* 8 Hz), 4.08 (1H, dd, *J* *12* *=* 12 Hz *, J* *13* *=* 16 Hz), 5.70 (1H, dd, *J* *12* *=* 8 Hz *, J* *13* *=* 12 Hz), 6.89 (2H, d, *J =* 8 Hz), 7.29 (2H, d, *J =* 8 Hz), 7.52 (2H, d, *J* *23* *=* 8 Hz), 7.50 (1H, d, *J =* 8 Hz), 7.59 (2H, d, *J =* 8 Hz), 7.72 (1H, m), 7.68 (1H, m), 7.87 (1H, s), 8.02 (1H, d, *J =* 8 Hz),13C NMR (100 MHz, CDCl3) δ (ppm) with TMS = 0: 148.5, 147.3, 147.1, 147.0, 147.3, 147.5, 147.7, 142.9, 135.9, 135.7, 135.6, 135.5, 135.9, 131.9, 131.8, 130.9, 128.3, 127.9, 127.6, 127.4, 123.4, 114.9, 112.1, 42.28, mass (EI): calculated for C24H16Br2ClN3: 538.94 gm/mol[M+]; found at 538, [M+2] peak at 541.

**3-(1-(4-bromophenyl)-5-(naphthalen-2-yl)-4,5-dihydro-1H-pyrazol-3-yl)-2-chloroquinoline (14)**

Greenish yellow crystals (yield: 75%), m.p.: 210–215 °C, 1H NMR [CDCl3, 400 MHz, δ (ppm) with TMS = 0]: 3.33 (1H, dd, *J* *12* *=* 8Hz *, J* *13* *=* 4 Hz), 4.25 (1H, dd, *J* *12* *=* 13 Hz *, J* *13* *=* 20 Hz), 5.75 (1H, dd, *J* *12* *=* 4 Hz *, J* *13* *=* 12 Hz), 6.97 (2H, m), 7.30 (2H, m), 7.45 (1H, m), 7.47 (1H, d, *J =* 4 Hz), 7.48 (1H, m), 7.69 (1H, m), 7.70 (1H, m), 7.80 (1H, m), 7.81 (1H, m), 7.82 (1H, m), 7.83 (1H, s), 7.92 (1H, s), 8.04 (1H, d, *J =* 8 Hz), 8.14 (1H, d, *J =* 8 Hz),13C NMR (100 MHz, CDCl3) δ (ppm) with TMS = 0: 148.68, 148.20, 147.32, 143.06, 135.98, 135.13, 133.74, 133.30, 131.98, 130.89, 129.87, 129.62, 128.50, 128.40, 128.27, 127.99, 127.92, 127.86, 127.62, 127.34, 126.85, 126.77, 125.81,125.10, 124.41, 123.39, 114.93, 111.91, mass (EI): calculated for C28H19BrClN3: 511.05 [M+]; found at 511, [M+2] peak at 513.

### 2.2. Molecular docking studies

The docking interactions were studied with the help of the Maestro v. 11.5 (Schrödinger Inc., New York, NY, USA) on a Mac workstation. The study was done to identify the possible binding interactions of selected ligands with the active site of the receptor. The ligand structures of the dataset were prepared by the LigPrep module of Maestro v. 11.5 [23,24]. Ligands were assisted by grid-based molecular docking to bind in more than one possible conformation. The protein structure was prepared using the protein preparation wizard (preprocessed, optimized, and minimized) in Maestro v. 11.5 [25]. The protonation and tautomeric states of amino acids were adjusted to match a pH of 7.4. Grid generation was done using the receptor grid generation module of Maestro v.11.5 [26]; 0.25 Å, scaling factor, and 1.0 Å, partial charge cut off for van der Waals radius were applied. Docking was carried out using XP (extra precision) mode. Glide molecular docking was utilized for the evaluation of binding interactions and ligand flexibility. Binding energies were calculated in kcal/mol. XP Visualizer was used for the analysis of the specific ligand–protein interactions. The compounds with the highest docking scores in negative and good interaction profiles were the most active compounds against the target receptor protein [27–29]. The 2D interaction diagrams of rilpivirine, elvitegravir, and designed compounds are mentioned in Table 4.

**Table 4:** Docking pose of synthesized compounds and their dock score.

Comp.	Docking pose	Docking score (Kcal/mol)
Elvitegravir(Reference)	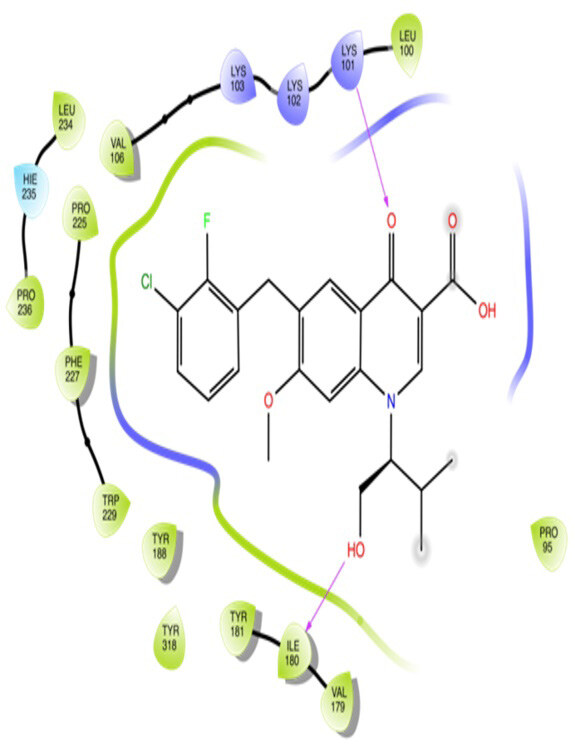	–8.57
Rilpivirine (Reference)	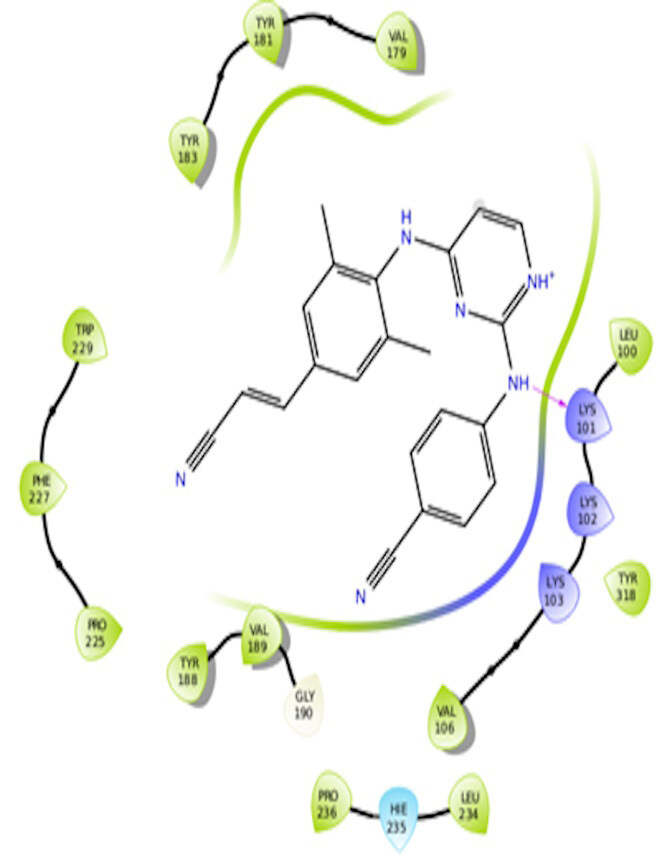	–8.56
4	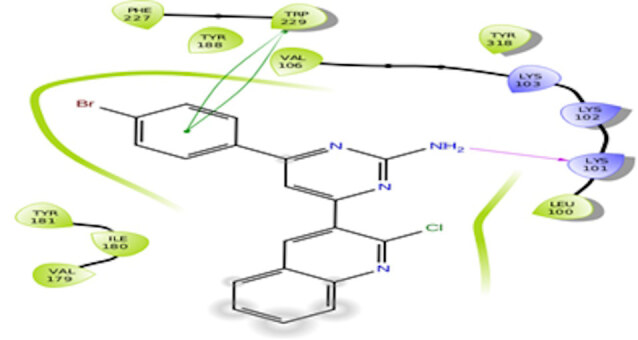	–10.67
5	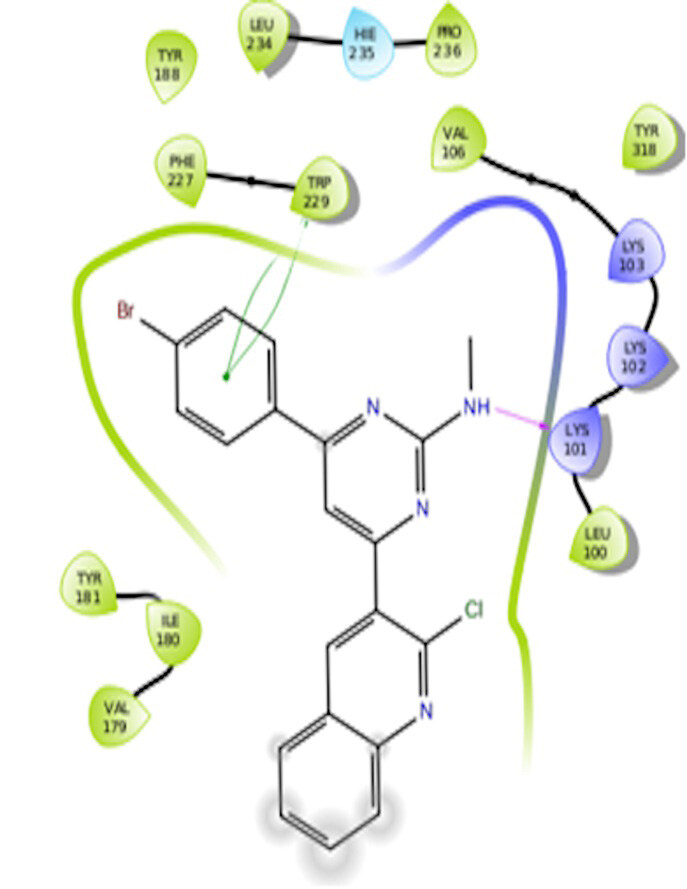	–10.38
6	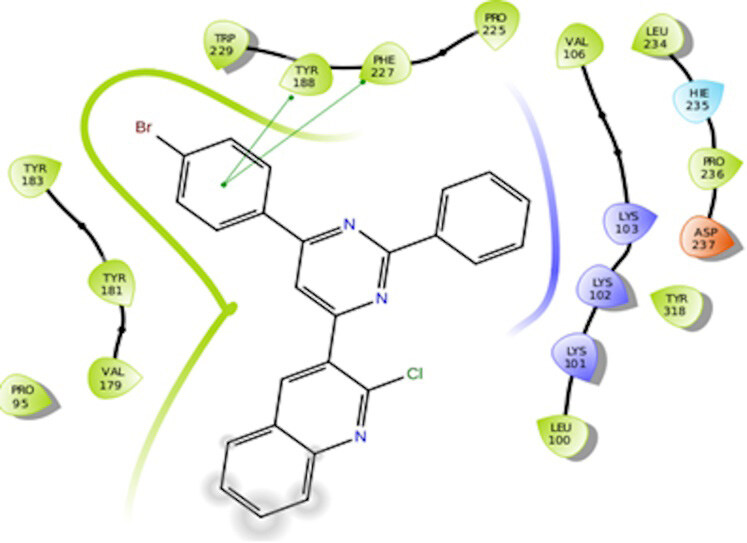	–10.19
7	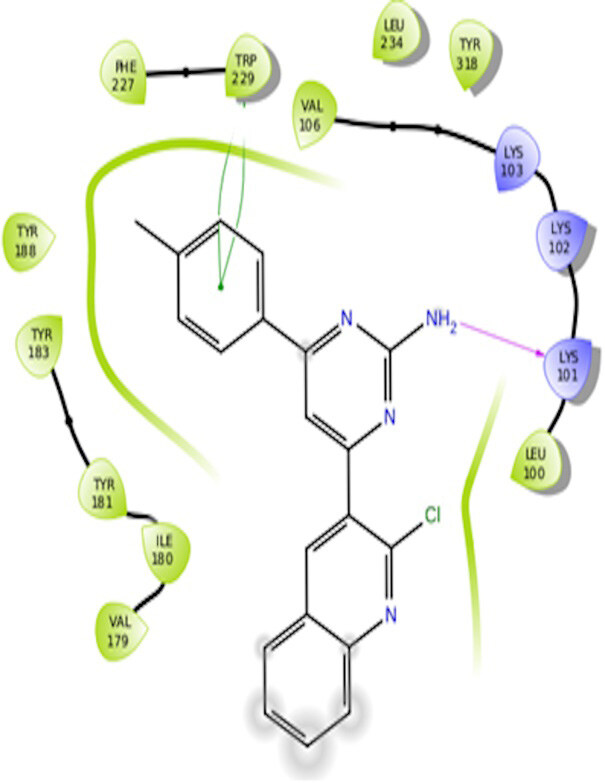	–10.23
8	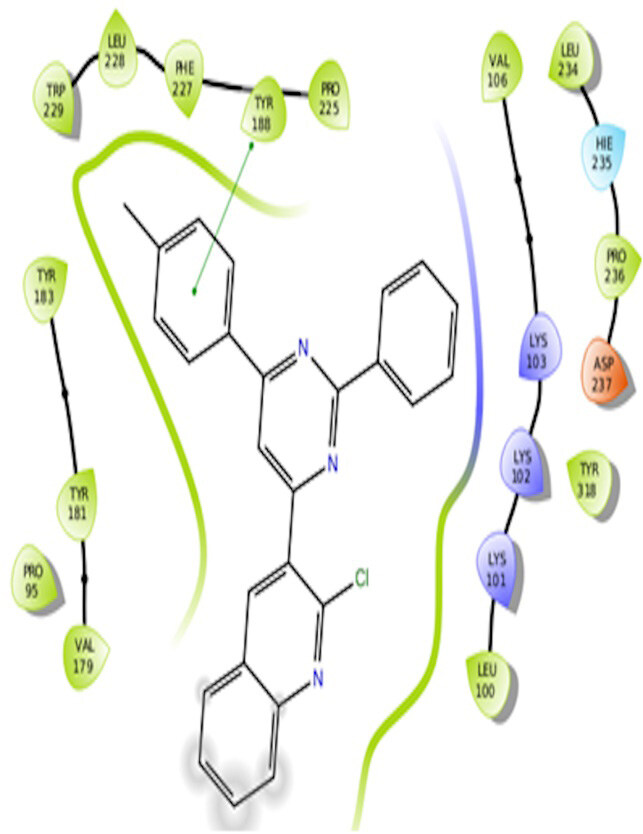	–9.96
9	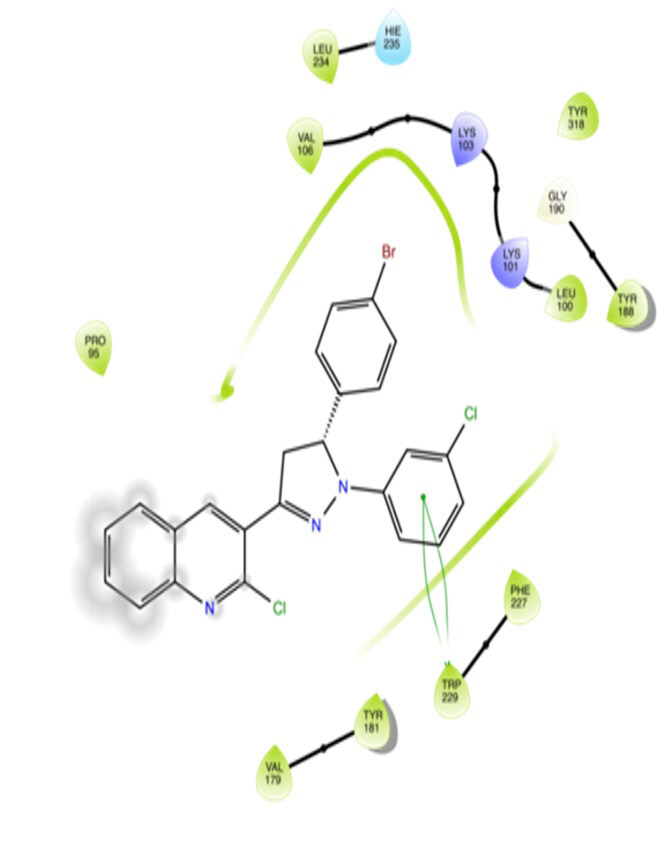	–7.93
10	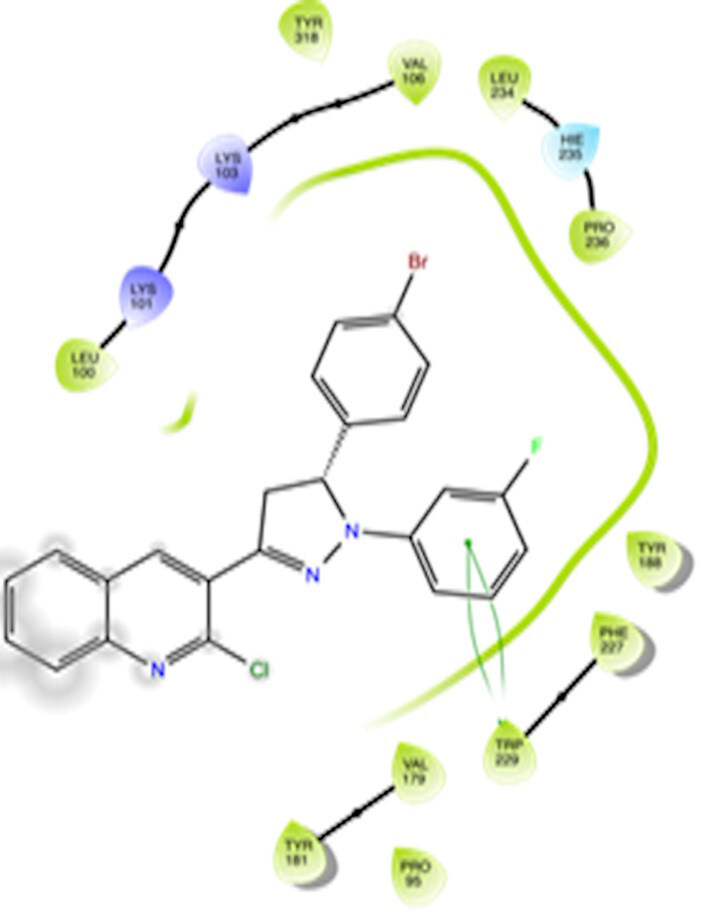	–7.75
11	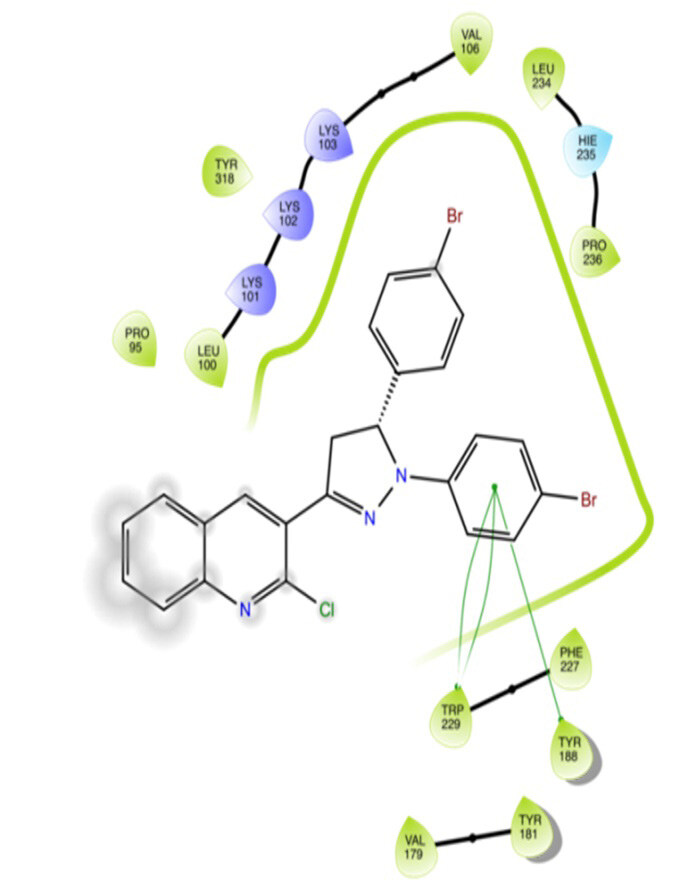	–8.76
12	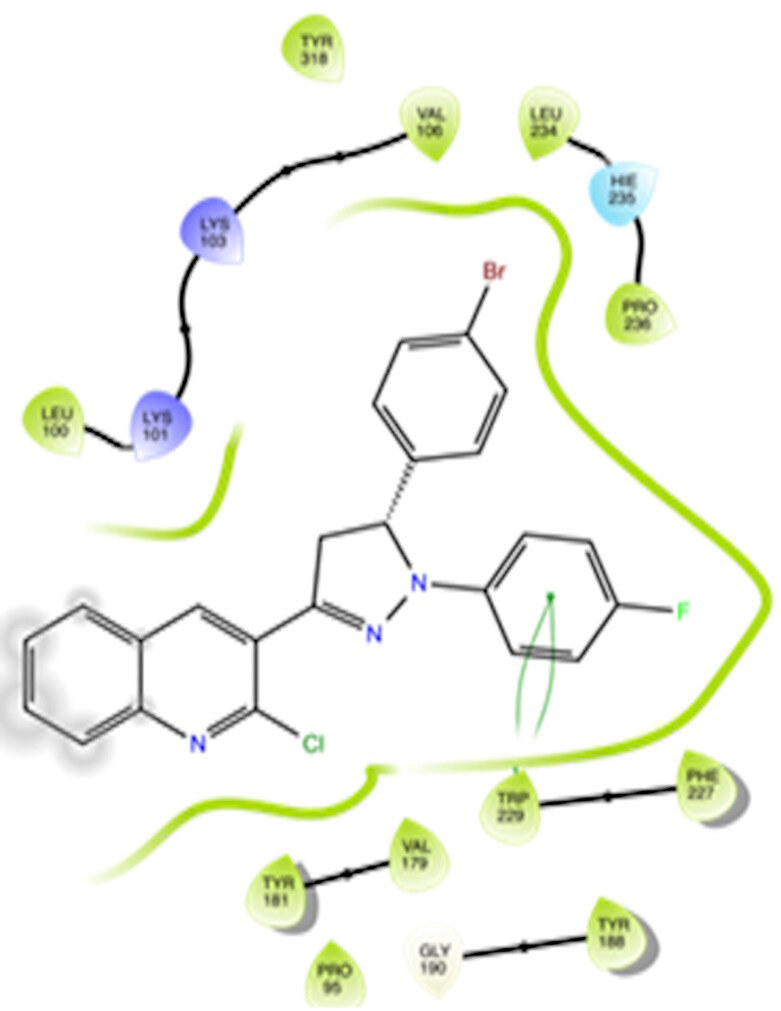	–8.51
13	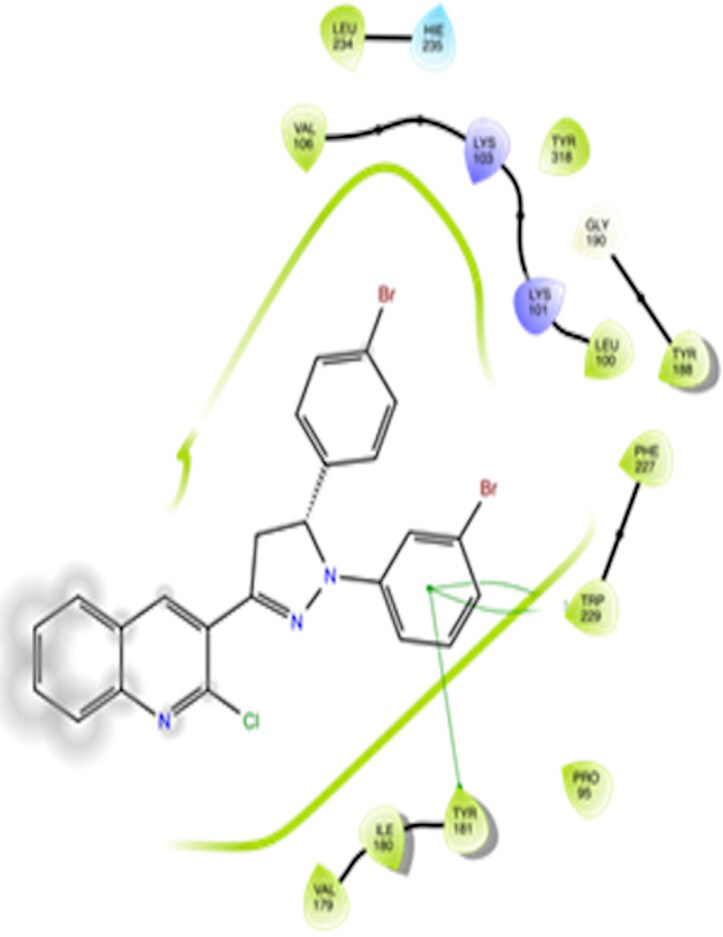	–8.08
14	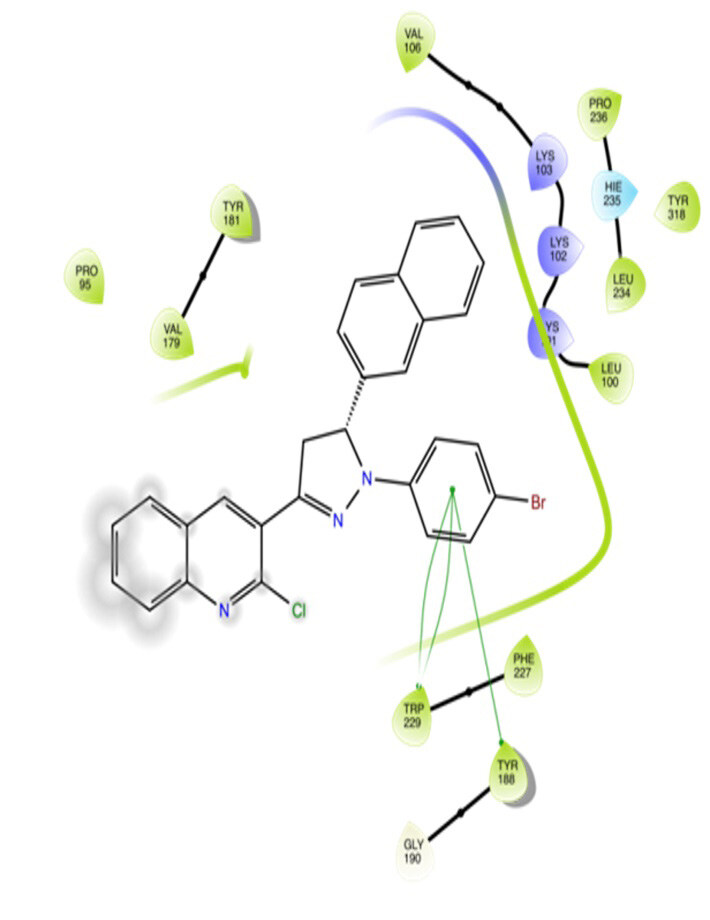	–9.34

## 3. Results and discussion

### 3.1. Synthesis of compounds

**Figure 1:**
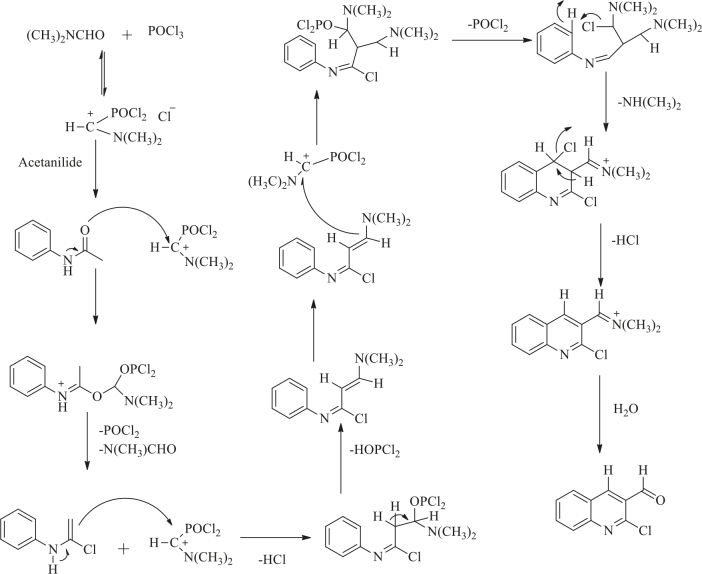
Reaction mechanism involved in synthesis of starting material.

The general chemistry of the synthesis of the starting material, 2-chloroquinoline-3-carbaldehyde (1), is based on the Vilsmeier–Haack reaction [30,31]. The N-phenylacetamide after formylation and cyclization formed 2-chloroquinoline-3-carbaldehyde. The Vilsmeier reagent acted as formylating agent (Scheme, Figure 1). Compound (1) and substituted acetophenones were taken as starting material for the synthesis of chalcones from which 2 series of compounds were synthesized.

**Scheme 1:**
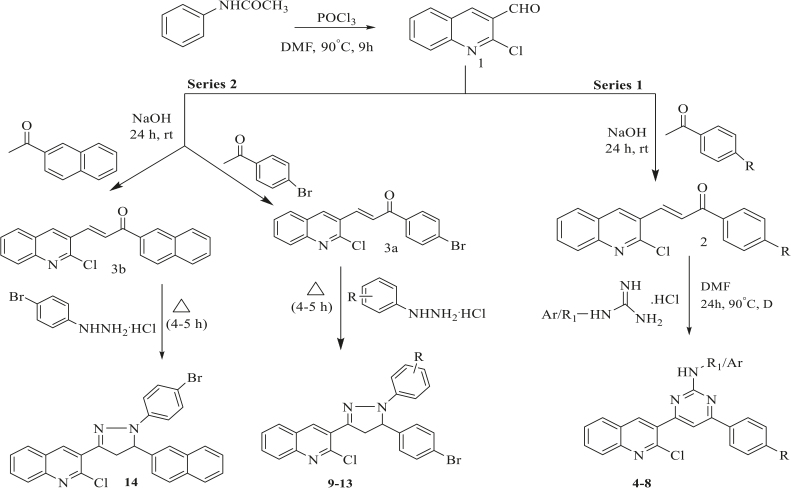
Scheme for synthesis of designed/proposed compounds.

**Figure 2:**
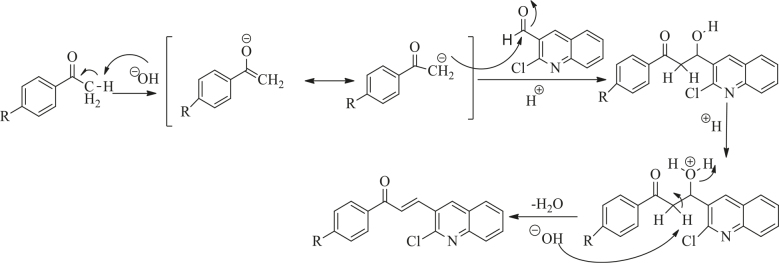
Reaction involved in synthesis of chalcone.

Chalcones were synthesized by the Claisen–Schmidt condensation reaction as described in Figure 2. Mechanistically, base-catalysed Claisen–Schmidt condensation was characterized by the formation of an enolate ion of the ketone [32]. This enolate ion acting as the nucleophile attacked the electrophilic carbon of the aldehyde, in the process rendering to the electron-rich ion, which took up the proton from the aqueous solution. The final step of the reaction was dehydration, which produced the chalcone.

**Figure 3:**
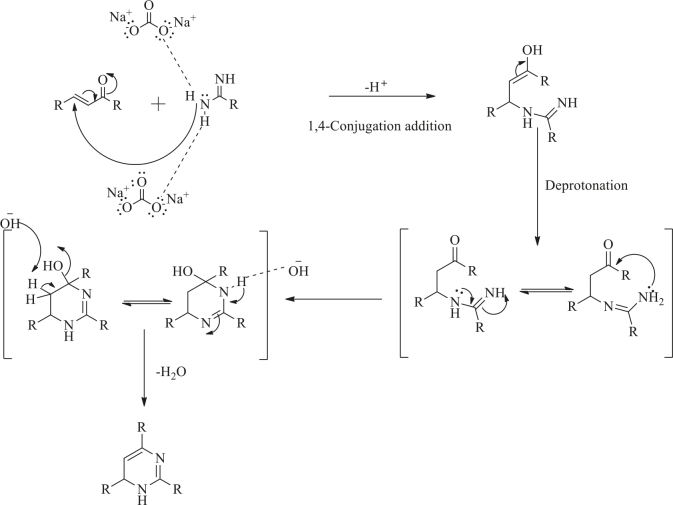
Reaction involved in synthesis of pyrimidine derivatives.

**Figure 4:**
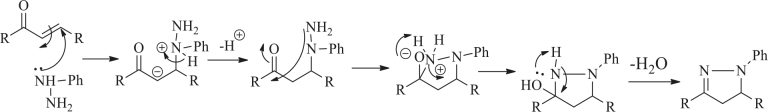
Reaction involved in synthesis of pyrazole derivatives.

In one of the series, pyrimidine derivatives were synthesized from chalcones using guanidine hydrochloride, benzamidine hydrochloride, and methyl guanidine hydrochloride. The reaction proceeded by Michael addition to the chalcones, followed by proton transfer, cyclization via Claisen addition, hydrolysis, and spontaneous dehydration, as described in Figure 3. In the other series, pyrazoline derivatives were synthesized from chalcones using substituted phenylhydrazine hydrochloride as shown in Figure 4.

### 3.2. Molecular docking studies

The docking studies were executed through the Maestro module (Schrödinger Inc.). Rilpivirine and elvitegravir were taken as the standard inhibitors of HIV-RT (PDB ID: 4I2P). The synthesized compounds were docked against rilpivirine, as it showed high binding affinity and docking score with the protein compared to other pyrimidine-containing NNRTIs. This PDB ID was selected as the resolution of the protein structure was good (2.30 Å) and it was in a complex with a rilpivirine (TMC278)-based analogue. We have redocked the cocrystallized ligand, namely (2E)-3-[4-({6-[(4-methoxyphenyl)amino]-7H-purin-2-yl}amino)-3,5-dimethylphenyl]prop-2-enenitrile within the same constraints as where it was crystallized with 4I2P; the docking score and RMSD value were recorded. The docking score was the same as previously calculated (–11.63 Kcal/mol), which showed that the process was precise; the RMSD value was 0.559, indicating the accuracy of the docking process.

**Figure 5:**
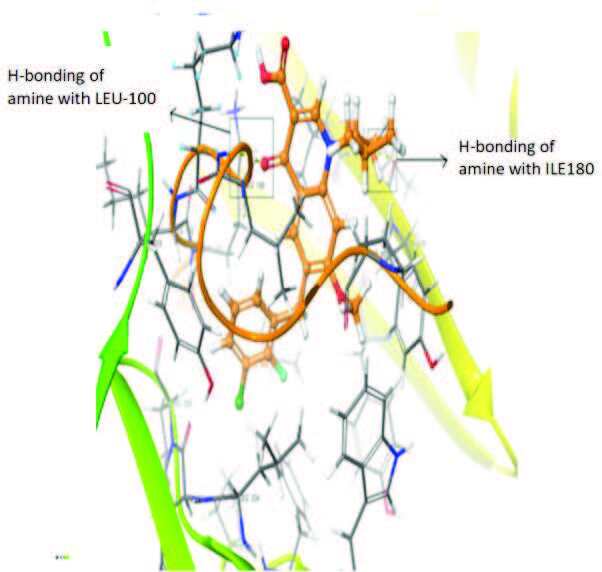
3D Interaction pattern of elvitegravir with reverse transcriptase protein (4I2P).

The docking profile demonstrated that all of the proposed ligands displayed a defined and strong binding affinity for the reverse transcriptase, as indicated by their docking score values. From 2D and 3D interaction diagrams of elvitegravir (Table 4, Figure 5), it was found that standard inhibitor binds to the target site; showed hydrogen bonding interaction with LYS 101, ILE-180, and LEU-100 and docking score (–8.57). The standard drug rilpivirine also showed hydrogen bonding with LYS 101; its docking score was –8.56 (literature value = –7.61) [33]. The designed compounds fit best in the allosteric site of HIV reverse transcriptase. Compounds 4–8 showed better docking scores than standard drugs (rilpivirine and elvitegravir), whereas the docking scores of designed compounds 11–13 were comparable with the standard drugs. In series 1, compounds 4, 5, and 7 exhibited the best docking scores (–10.67, –10.38, and –10.23, respectively) and displayed hydrophobic interactions with TRP229 and hydrogen bond interactions with LYS 101. Compounds 6 and 8, containing a phenyl ring attached to the phenyl amino group, exhibited slightly high docking scores (–10.19 and –9.96, respectively). Compound 6 showed hydrophobic interactions with TYR 188 and PHE 227, whereas compound 8 showed hydrophobic interactions with TYR 188 only. The lower docking score of compound 6 compared to compound 8 was due to the presence of a p-Br-phenyl moiety in compound 6 in place of a p-tolyl moiety at the fourth position of the pyrimidine ring. In series 2, the only member (compound 14) containing a naphthalene ring attached to the fifth position of the dihydropyrazole ring had the best docking score (–9.34), which was lower than those of both standard drugs; this compound showed hydrophobic interactions with TYR 188 and TRP 229. Compound 11 demonstrated pi-pi interactions witha docking score (–8.76) which was lower than those of both standard drugs. Compound 12 exhibited a docking score (–8.51) comparable to those of both standard drugs. The only difference between compounds 11 and 12 was the presence of p-Br-phenyl and p-F-phenyl in these compounds, respectively. The result suggested that compounds 9, 10, and 13 came up with higher docking score values than both standard drugs.

## 4. Structure–activity relationship

Based on the docking results of the synthesized compounds, the following SAR may be proposed:

· In the case of the pyrimidine derivatives (series 1), substitution of the R at para position of the phenyl ring with the electron-withdrawing bromo group gave better binding affinity compared to those with an electron-releasing methyl group.

· The free amino group attached to the pyrimidine ring was favourable for activity due to H-bond interactions. The substitution of this amino group with a methyl group resulted in a decrease in binding affinity, which was further reduced by substitution with a phenyl ring.

· In the case of pyrazoline derivatives (series 2), the substitution of R1 with a naphthalene ring gave better results compared to that with a *p-* Br *-* phenyl ring.

· Substitution of R with halogen atoms at the *p* -position gave better results compared to those at *m* -position, and substitution with the bromo group gave better activity compared with chloro and fluoro groups

**Figure 6:**
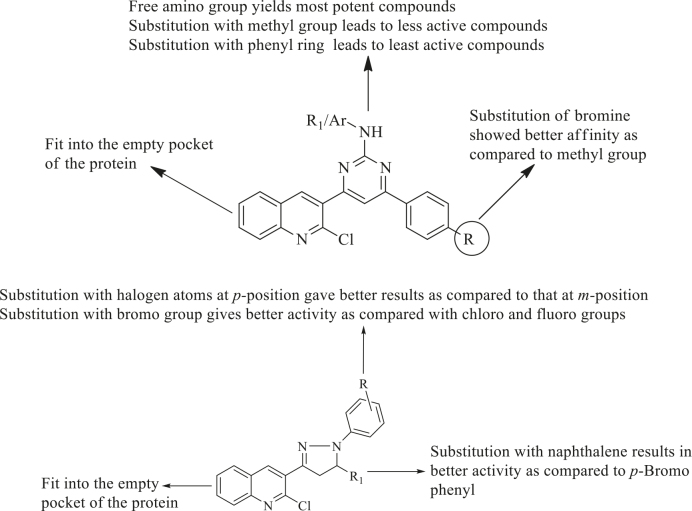
SAR of synthesized compounds.

· Quinoline moiety occupied the empty pocket in the binding cavity of the enzyme in the case of both pyrimidine and pyrazoline derivatives (series 1 and series 2). The abovementioned findings are summarized in Figure 6.

## 5. Conclusion

Quinoline derivatives were designed concerning the structures of standard anti-HIV drugs and synthesized via chalcones as intermediates. The synthesized compounds were characterized by physicochemical and spectral means. Molecular docking studies were performed on the synthesized compounds for their HIV reverse transcriptase inhibitory activity by using elvitegravir and rilpivirine as standard drugs; promising results were obtained. Compound 4 was found to have the highest affinity towards the reverse transcriptase protein (docking score = –10.67). In general, quinoline derivatives containing pyrimidine moiety (4–8) showed higher docking scores than those possessing pyrazoline moiety (9–14). SAR studies of the synthesized compounds were also performed based on their docking scores, which revealed that in the case of pyrimidine derivatives, a phenyl ring with an electron-withdrawing bromo group and a free amino group (attached to the pyrimidine ring) showed better binding affinity. In the case of pyrazoline derivatives, naphthalene attached to the pyrazoline ring showed better results compared to the p-Br-phenyl ring. Substitution of the phenyl ring attached to the N atom of the pyrazoline ring with halogen atoms at p-position showed better results compared to that at the m-position; substitution with the bromo group showed better binding affinity than chloro and fluoro groups. Thus, based on the docking results, we can say that the synthesized compounds have the potential to serve as important leads in the discovery of new NNRTIs.

Supplementary Materials Click here for additional data file. Supplementary data associated with the article can be found in the online version, where all IR, NMR, and mass spectra related to the synthesized compounds have been provided.
