# Supplementary material for: Synthesis and molecular docking studies of quinoline derivatives as HIV non-nucleoside reverse transcriptase inhibitors
Source: Turk J Chem. 2020 Dec 16;44(6):1623–41. doi: 10.3906/kim-2004-14 (PMC7772092; doi:10.3906/kim-2004-14)
Supplement: Supplementary file 1 — Supplementary Materials [file turkjchem-44-1623-sup001.pdf]

Supplementary informations:

Synthesis and molecular docking studies of quinoline derivatives as HIV non-nucleoside reverse transcriptase inhibitors

Nivedita BHARDWAJ[1,3*], Diksha CHOUDHARY[1*], Akashdeep PATHANIA[1], Somesh BARANWAL[2], Pradeep KUMAR[1**]

[1]Department of Pharmaceutical Sciences and Natural Products, Central University of Punjab, Bathinda, India

[2]Department of Microbiology, Central University of Punjab, Bathinda, India

[3]Department of Pharmaceutical Engineering and Technology, Indian Institute of Technology (Banaras Hindu University), Varanasi, India

[*]Equal contribution; [**]**Correspondence:** pradeepyadav27@gmail.com

The spectroscopic data of all the synthesized compounds from starting material, intermediates and final compound are mentioned here. The data contains mass spectra, IR spectra , proton NMR and 13 C NMR spectra of the designed compounds.

Starting material : Compound 1:- 2-chloroquinoline-3-carbaldehyde

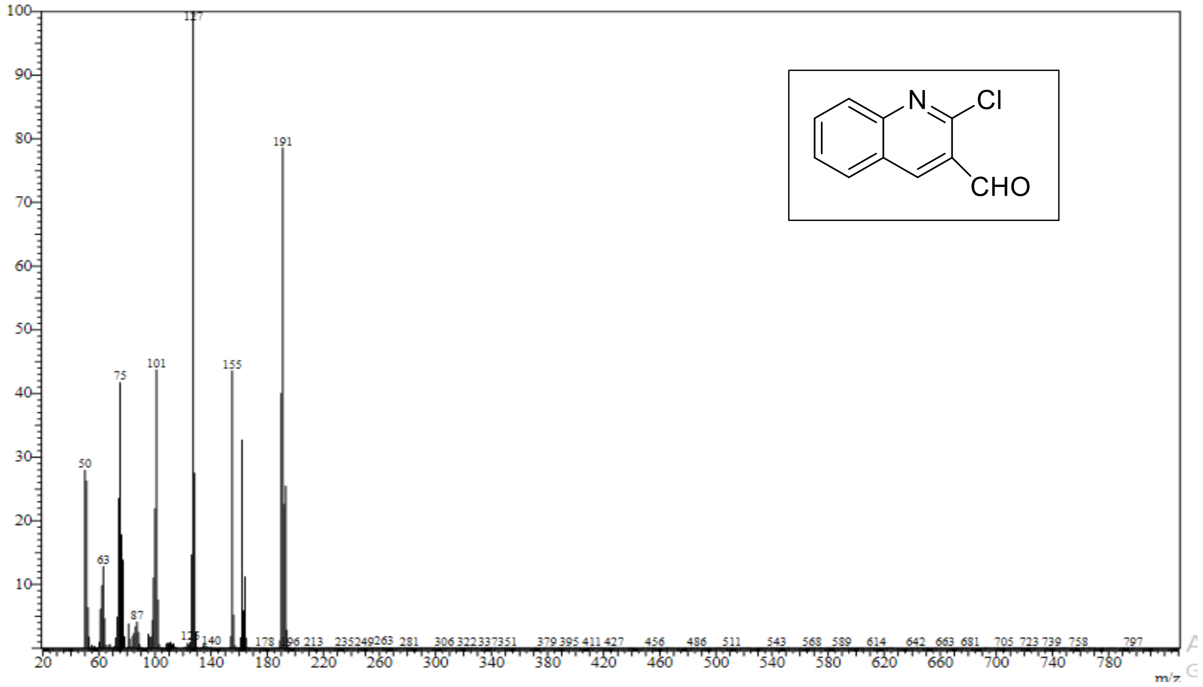

Mass spectrum of Compound 1

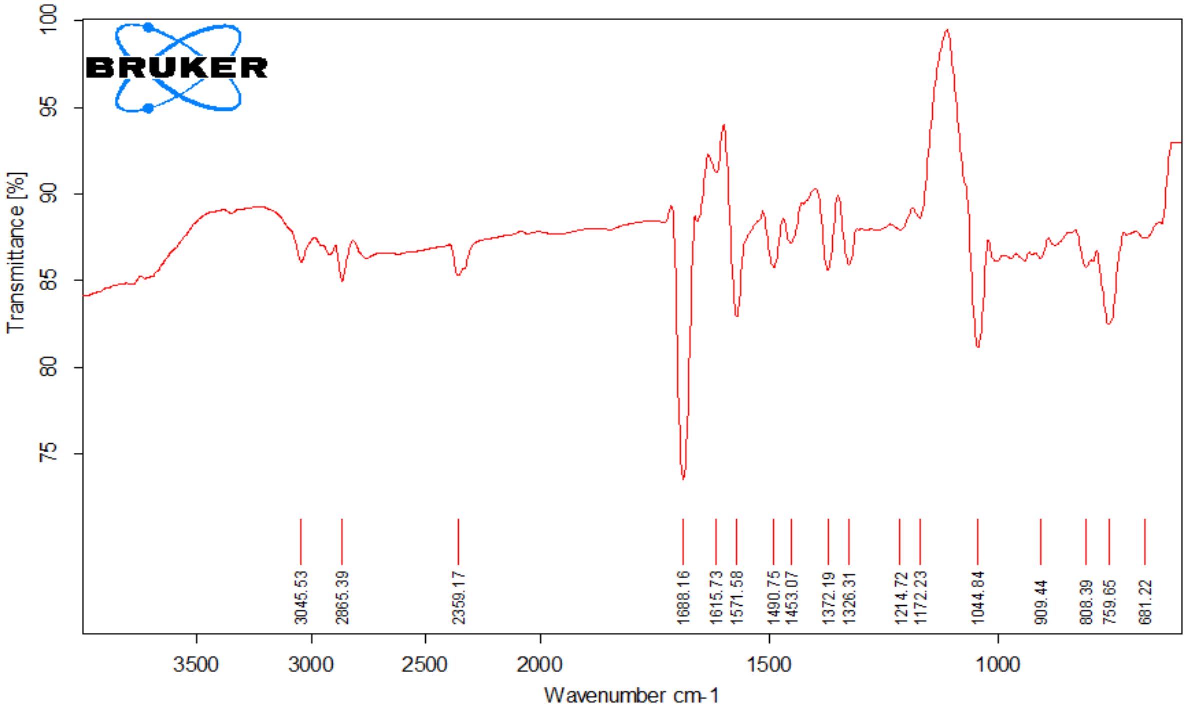

IR Spectrum of Compound 1

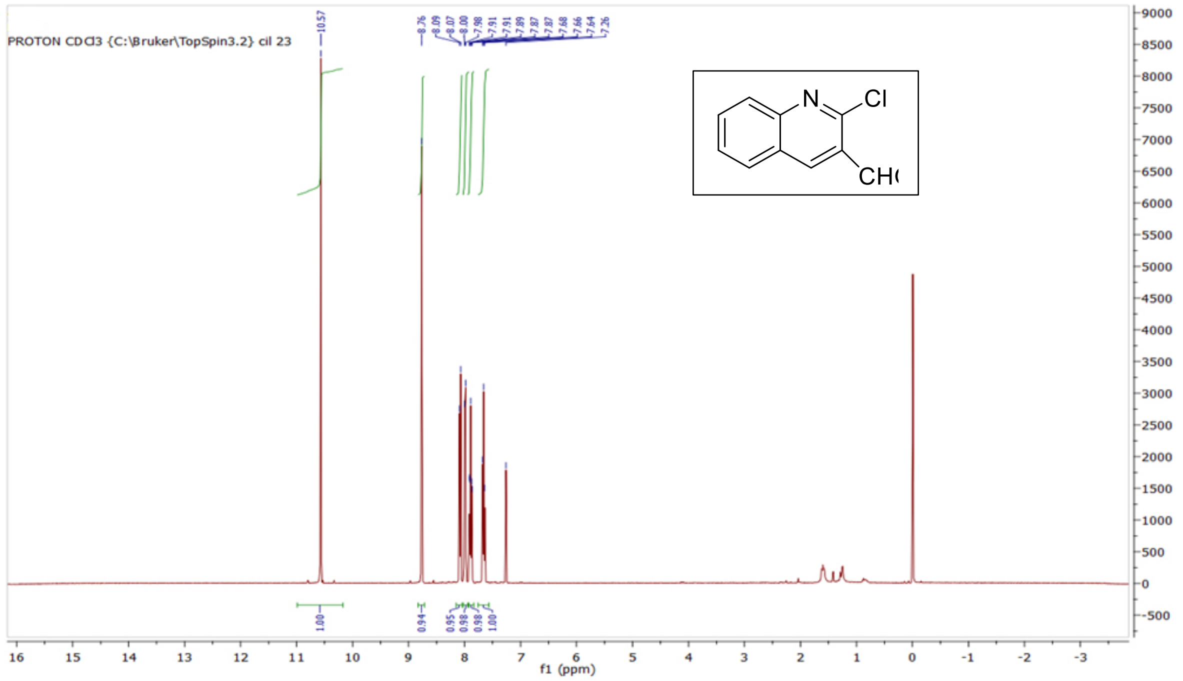

Proton NMR spectrum of Compound 1

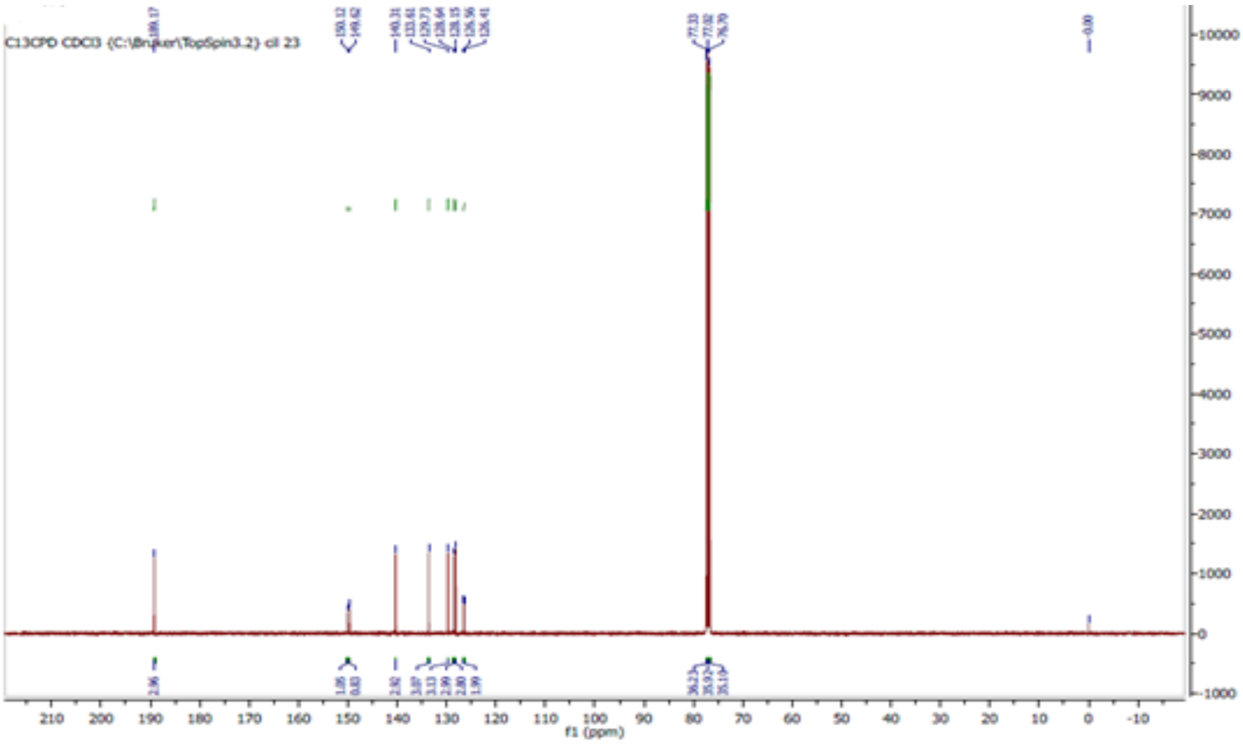

^{13}C NMR spectrum of Compound 1

Chalcones :

Compound 2 :- (E)-1-(4-bromophenyl)-3-(2-chloroquinolin-3-yl)-1- prop-en-1-one

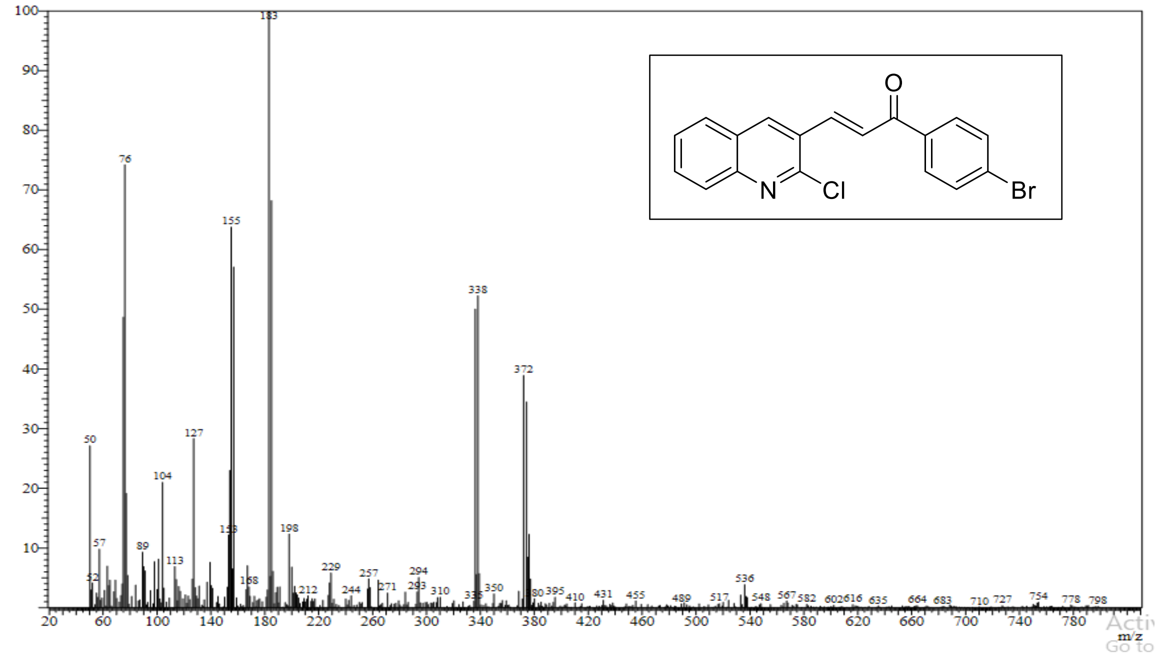

Mass spectrum of Compound 2

Compound 3:- (E)-3-(2-chloroquinolin-3-yl)-1-(p-tolyl)prop-2-en-1-one

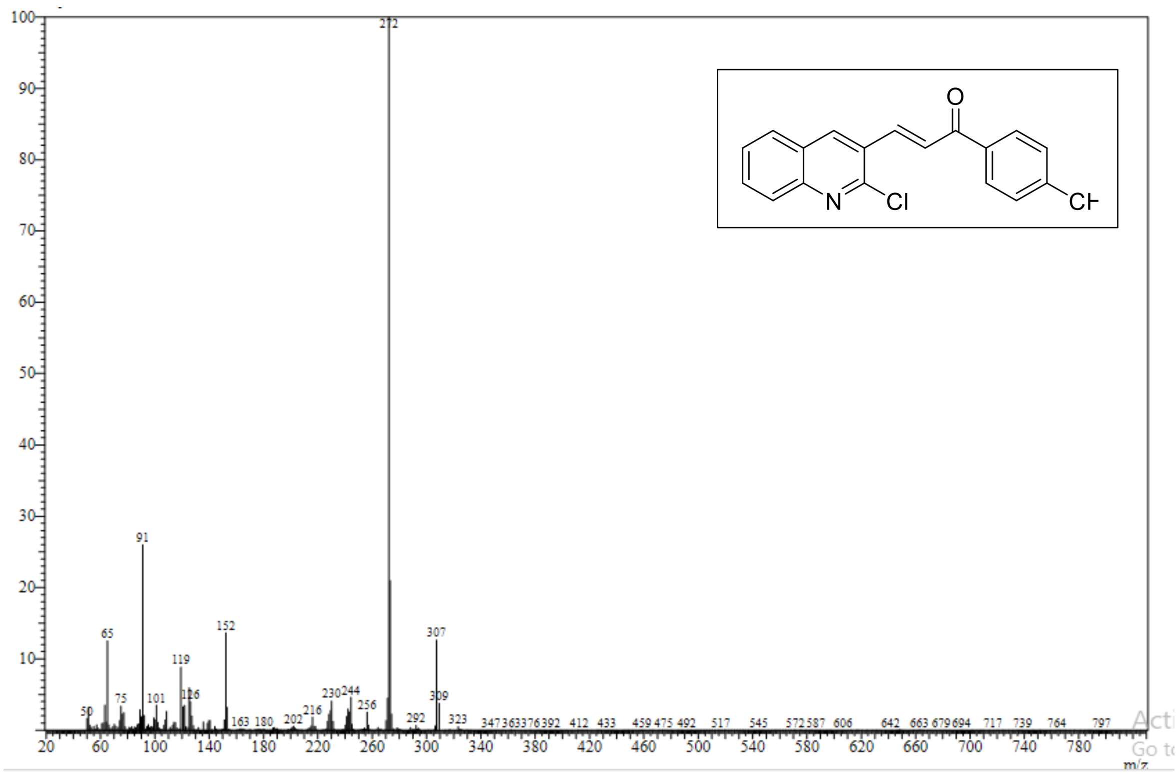

Mass spectrum of Compound 3

Final compounds:

Compound 4:- 4-(4-bromophenyl)-6-(2-chloroquinoline-3-yl)pyrimidin-2amine

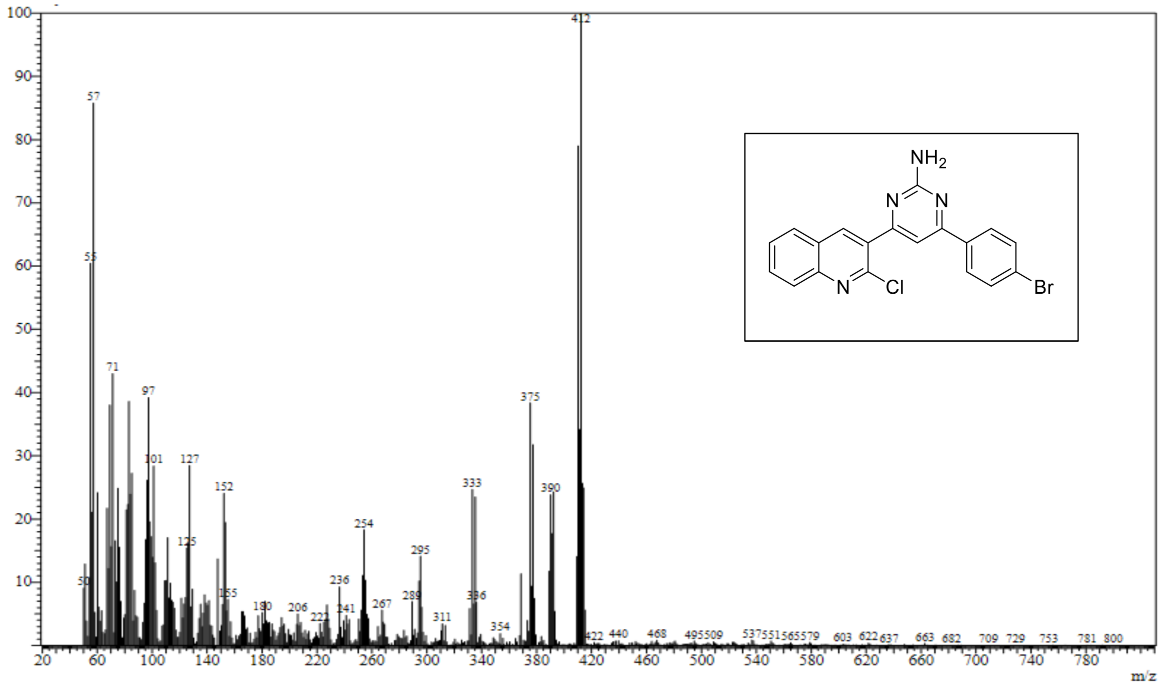

Mass spectrum of compound 4

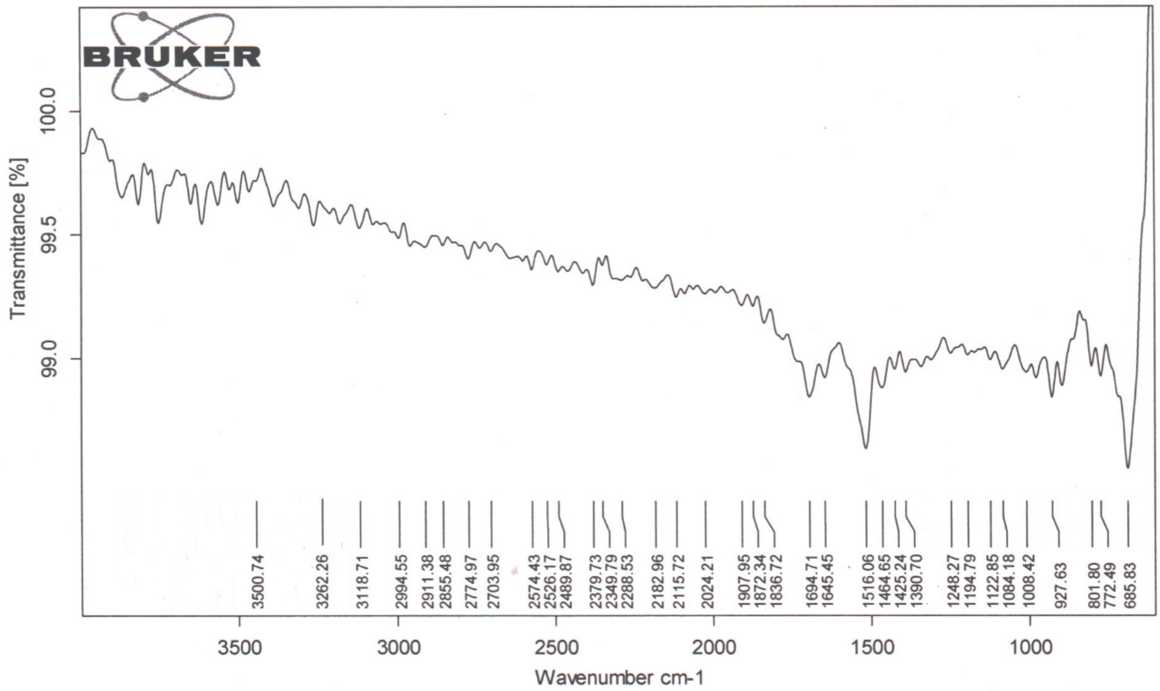

IR spectrum of compound 4

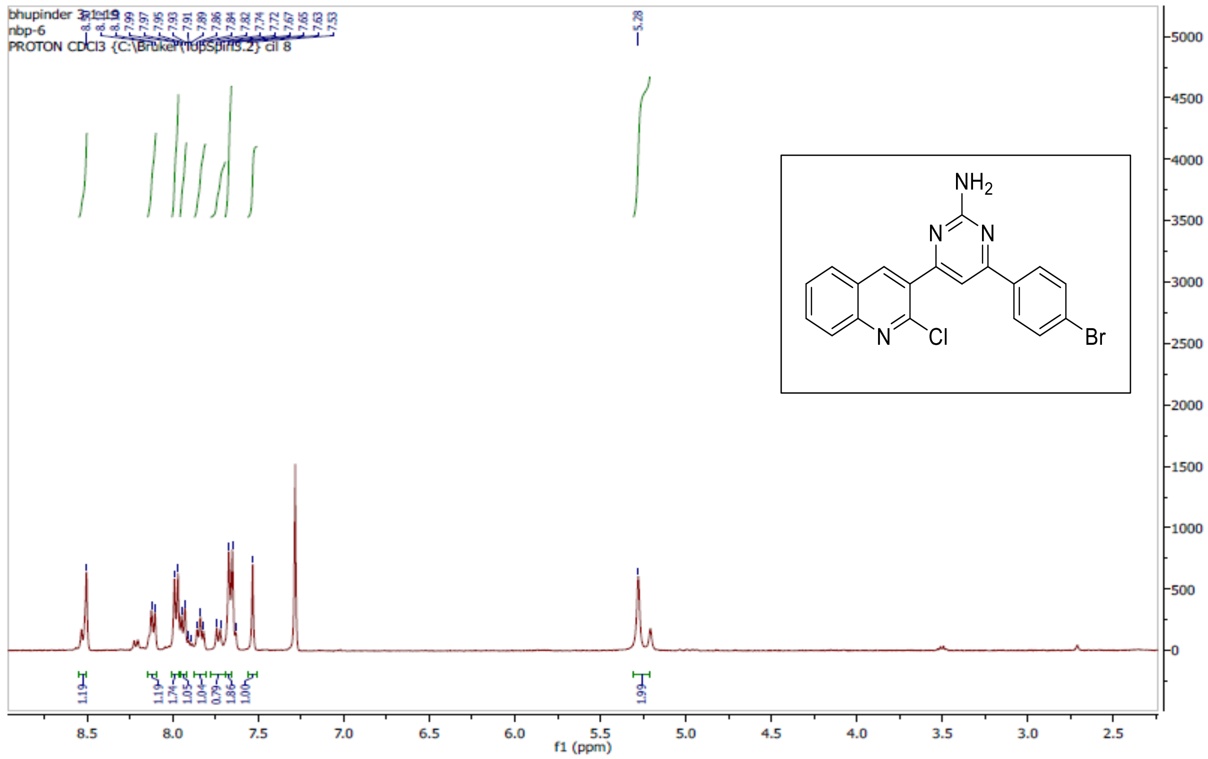

Proton NMR spectrum of compound 4

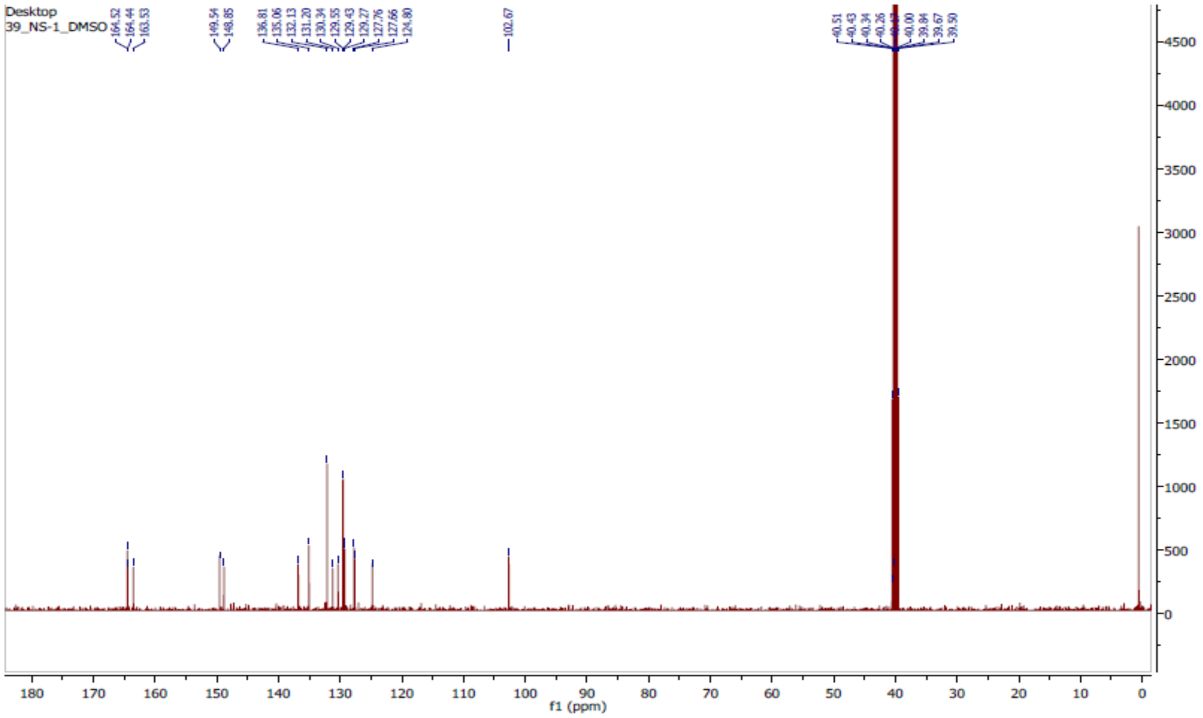

^{13} C NMR spectrum of compound 4

Compound 5:- 4-(4-bromophenyl)-6-(2-chloroquinoline-3-yl)-N-methylpyrimidin-2amine

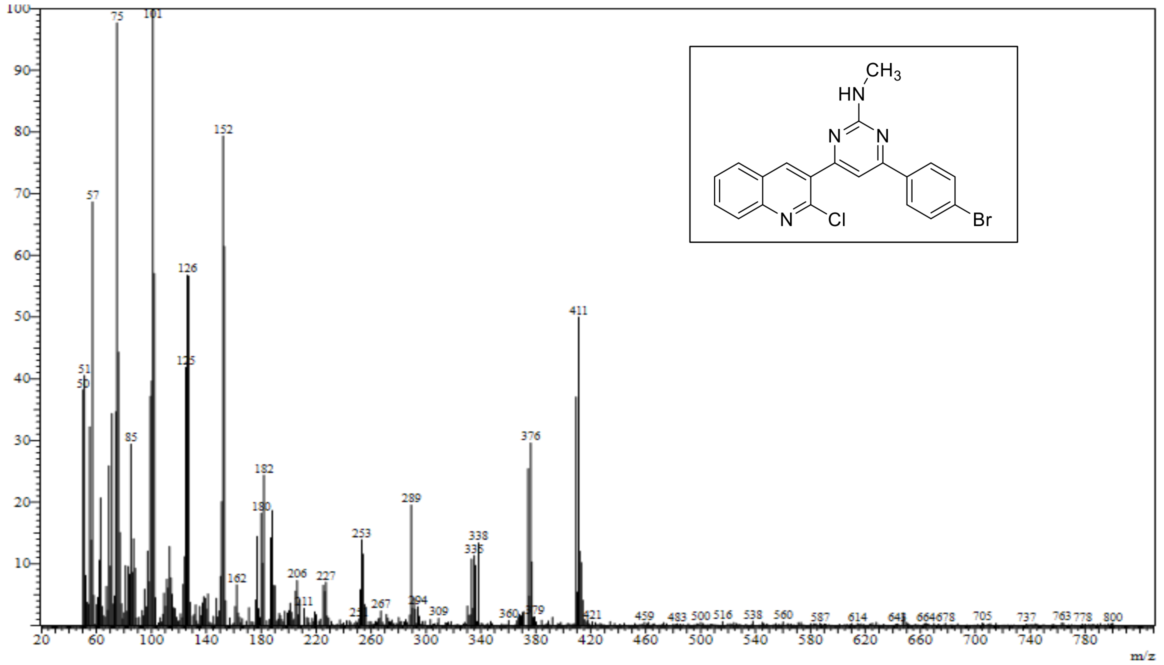

Mass spectrum of Compound 5

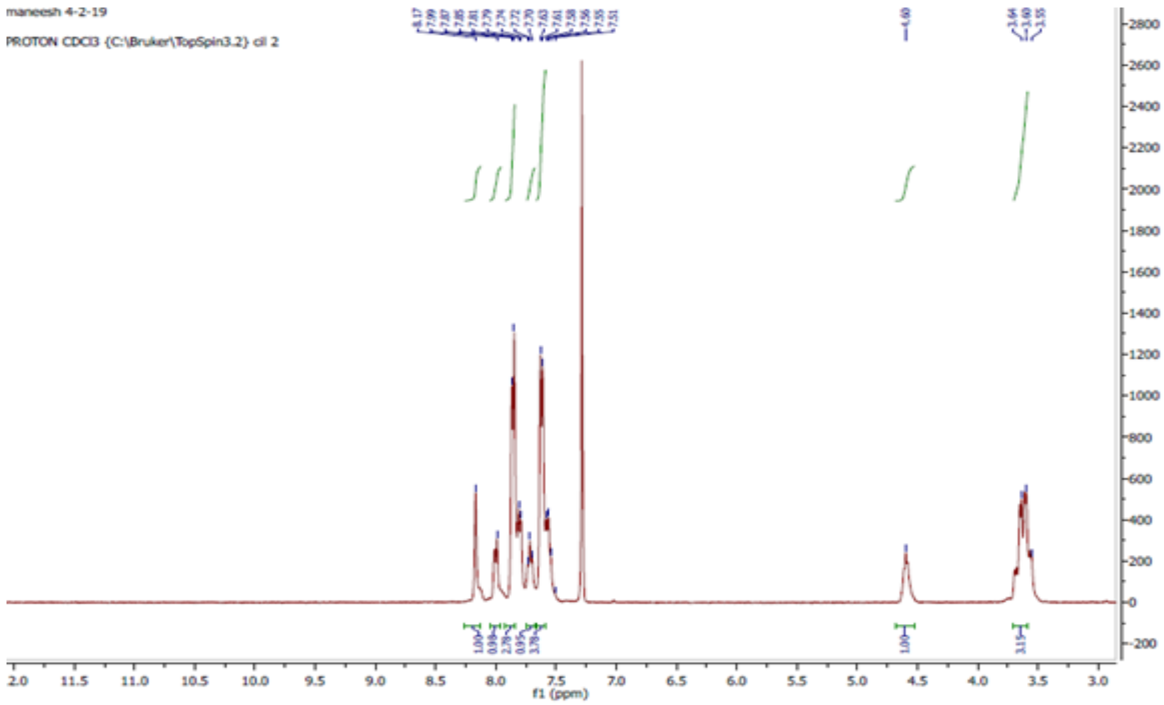

Proton NMR spectrum of compound 5

Compound 6: 4-(4-bromophenyl)-6-(2-chloroquinoline-3-yl)-N-phenylpyrimidin-2-amine

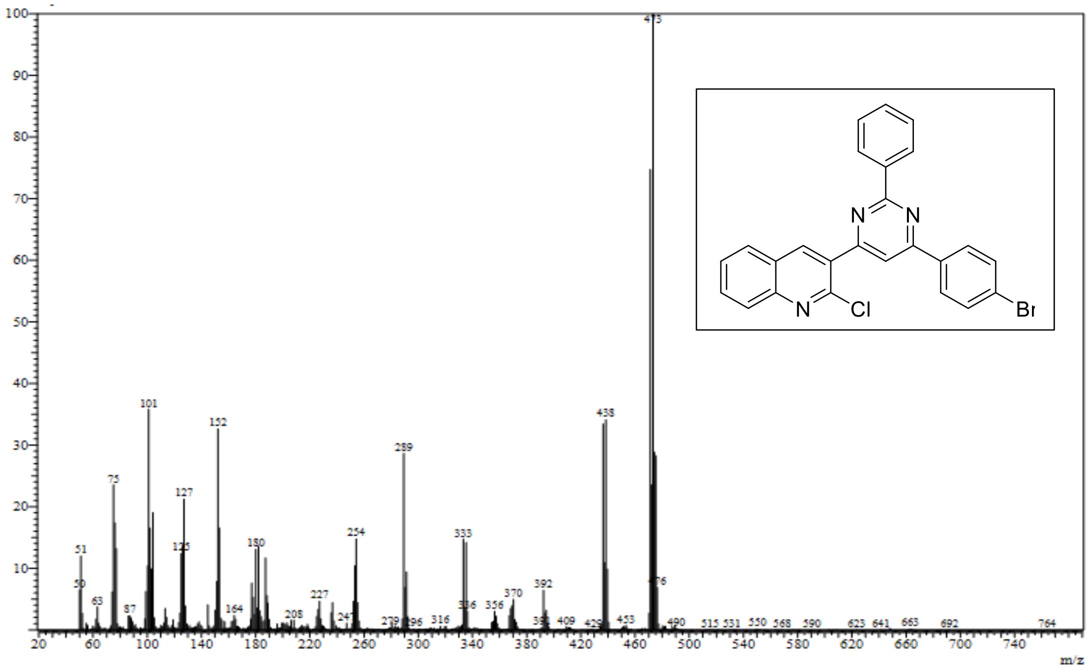

Mass spectrum of compound 6

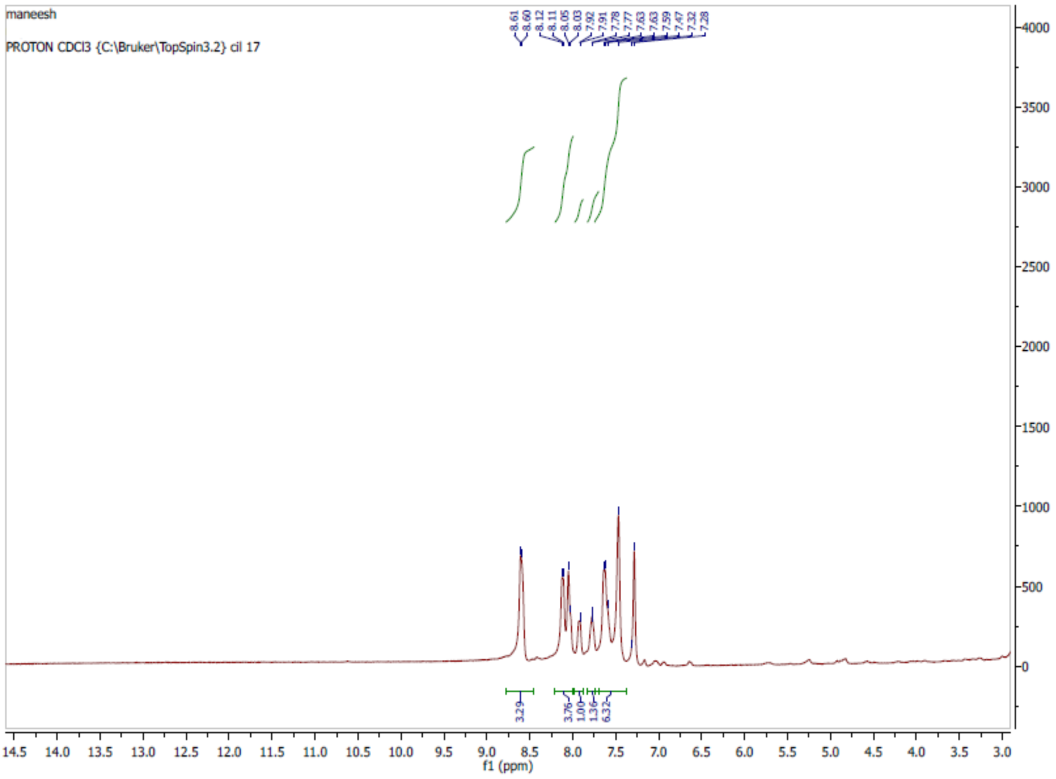

Proton NMR spectrum of compound 6

Compound 7:- 4-(4-bromophenyl)-6-(2-chloroquinoline-3-yl)-N-phenylpyrimidin-2-amine

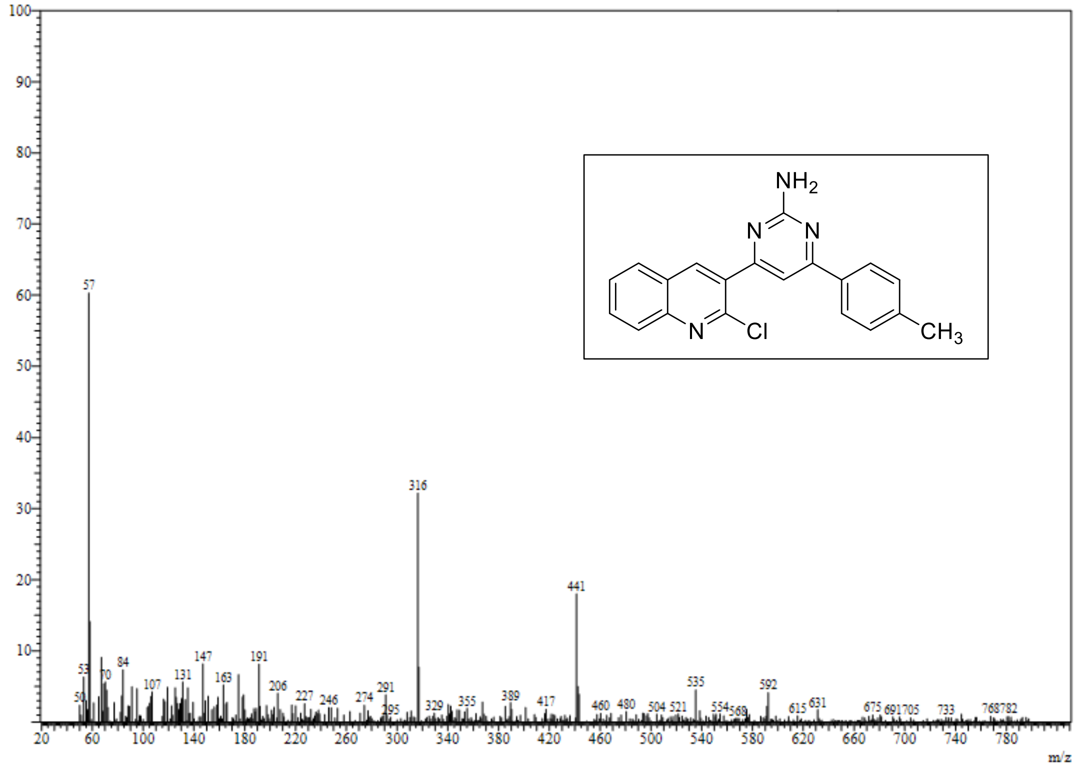

Mass spectrum of Compound 7

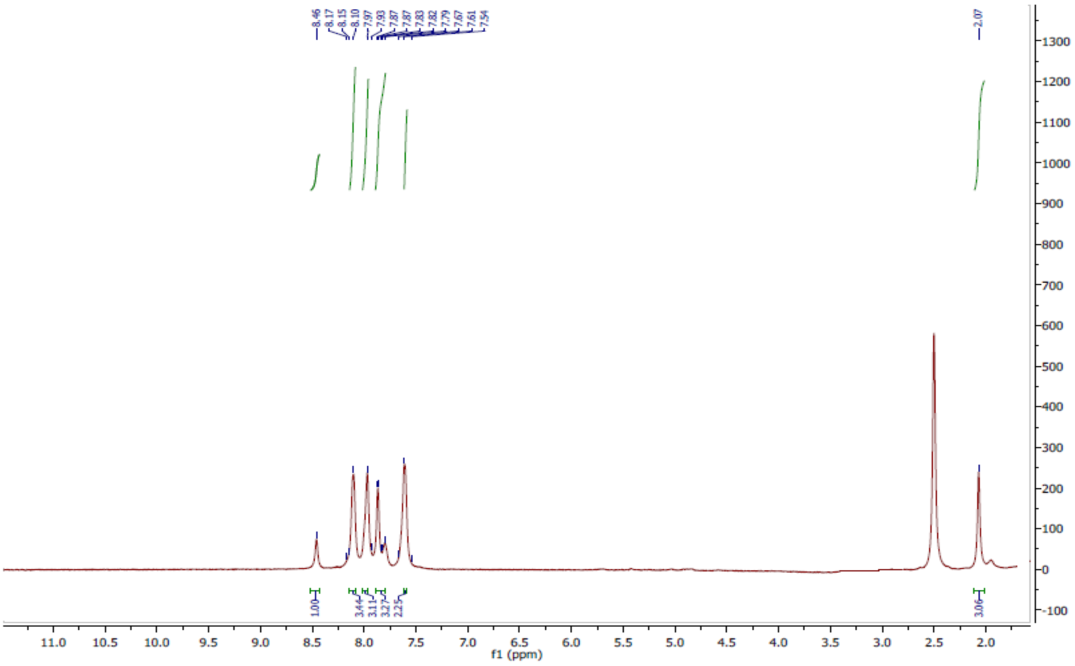

Proton NMR spectrum of compound 7

Compound 8:- 2-chloro-3-(2-phenyl-6-(p-tolyl)pyrimidin-4-yl)quinoline

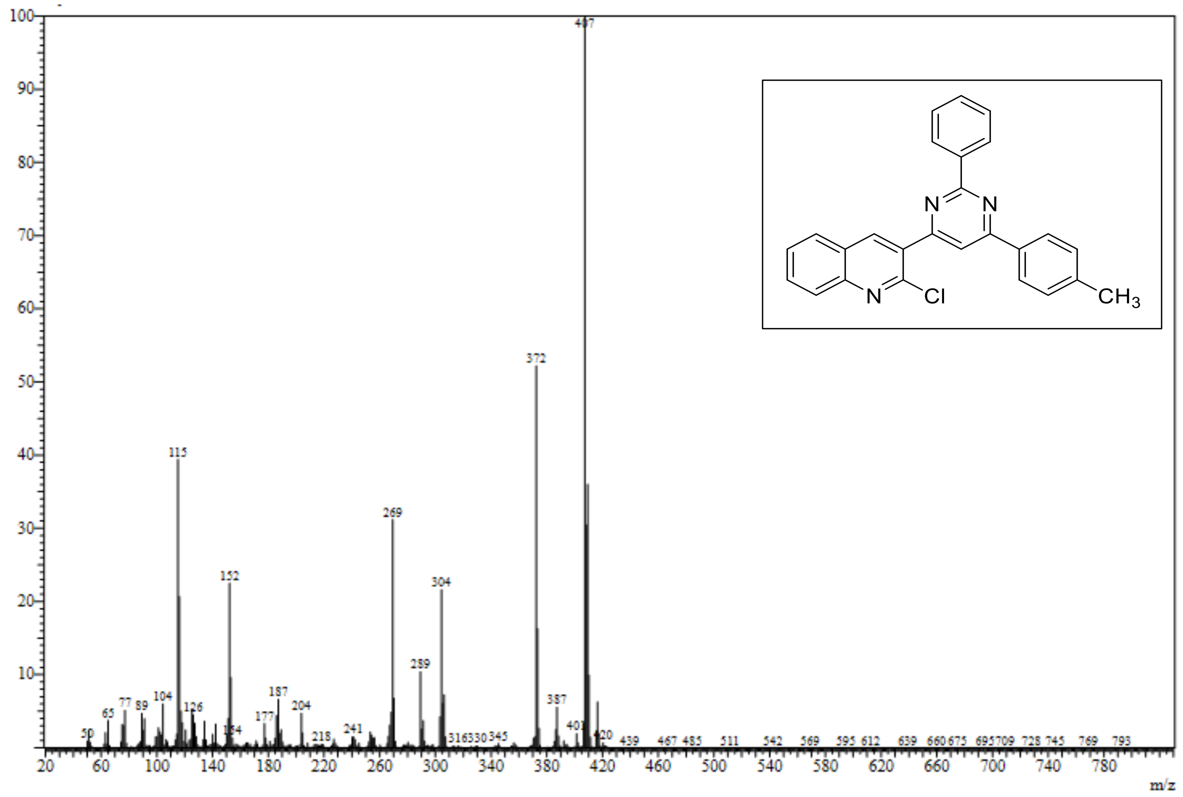

Mass spectrum of compound 8

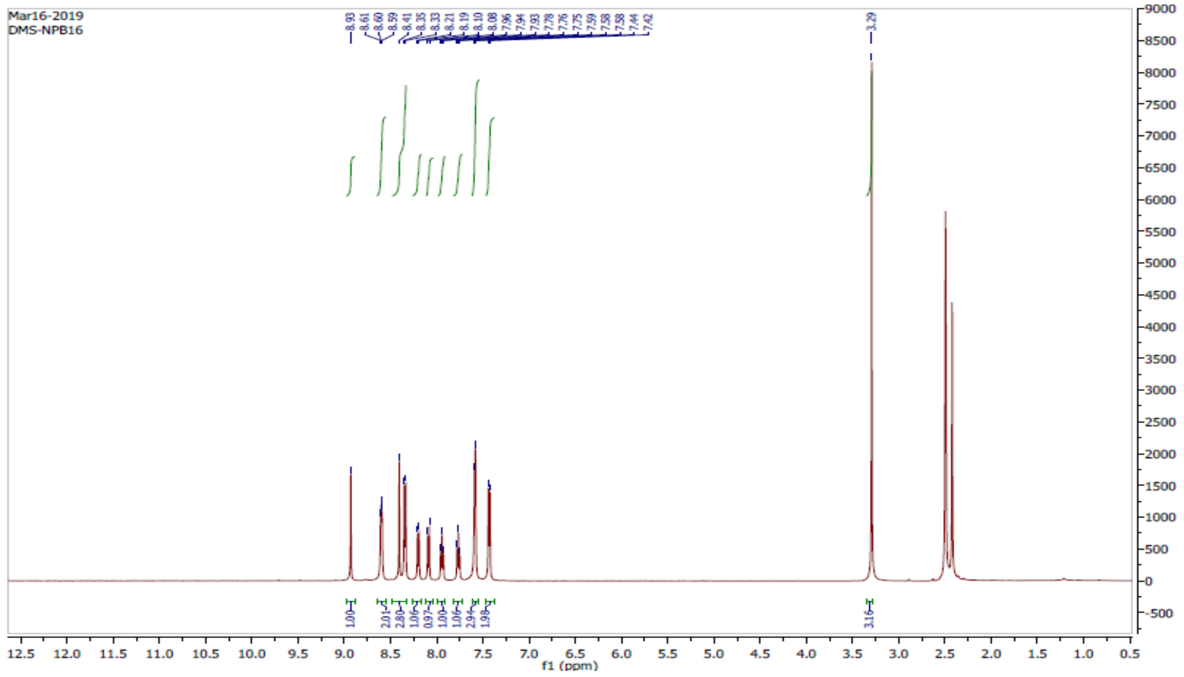

Proton NMR spectrum of compound 8

Compound 9:- 3-(5-(4-Bromophenyl)-1-(3-chlorophenyl)-4,5-dihydro-1H-pyrazol-3-yl)-chloroquinoline

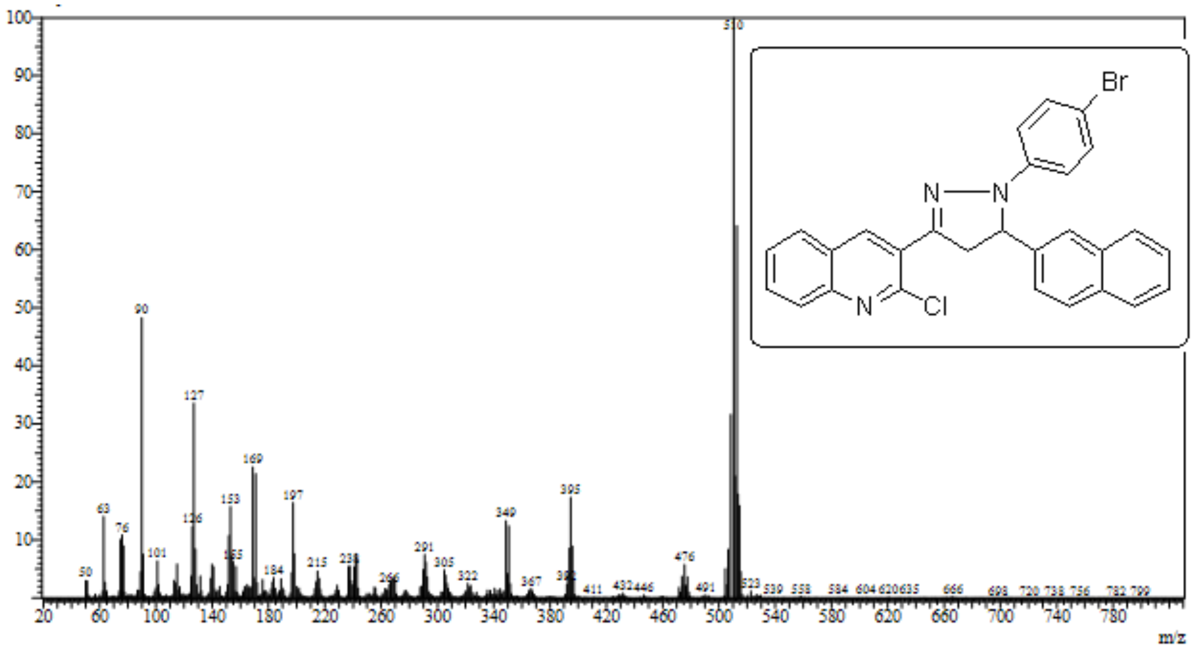

Mass spectrum of compound 9

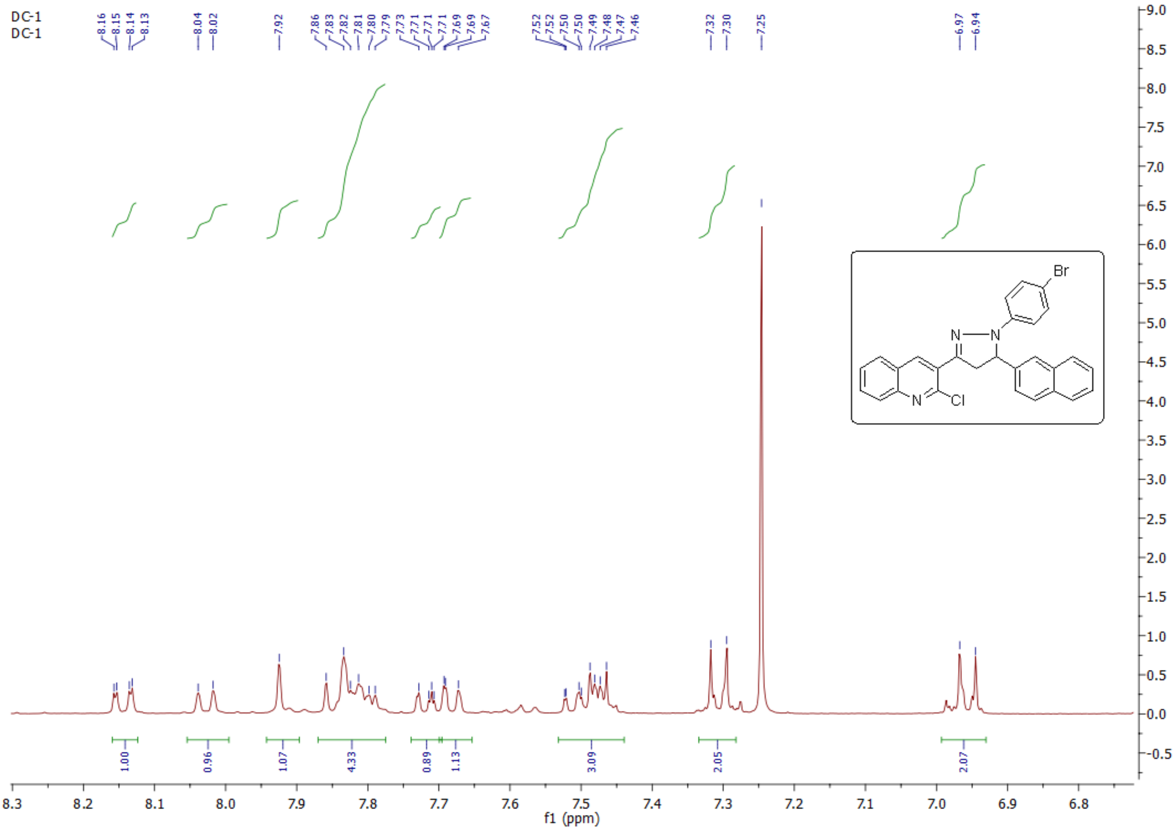

Proton NMR spectrum of compound 9

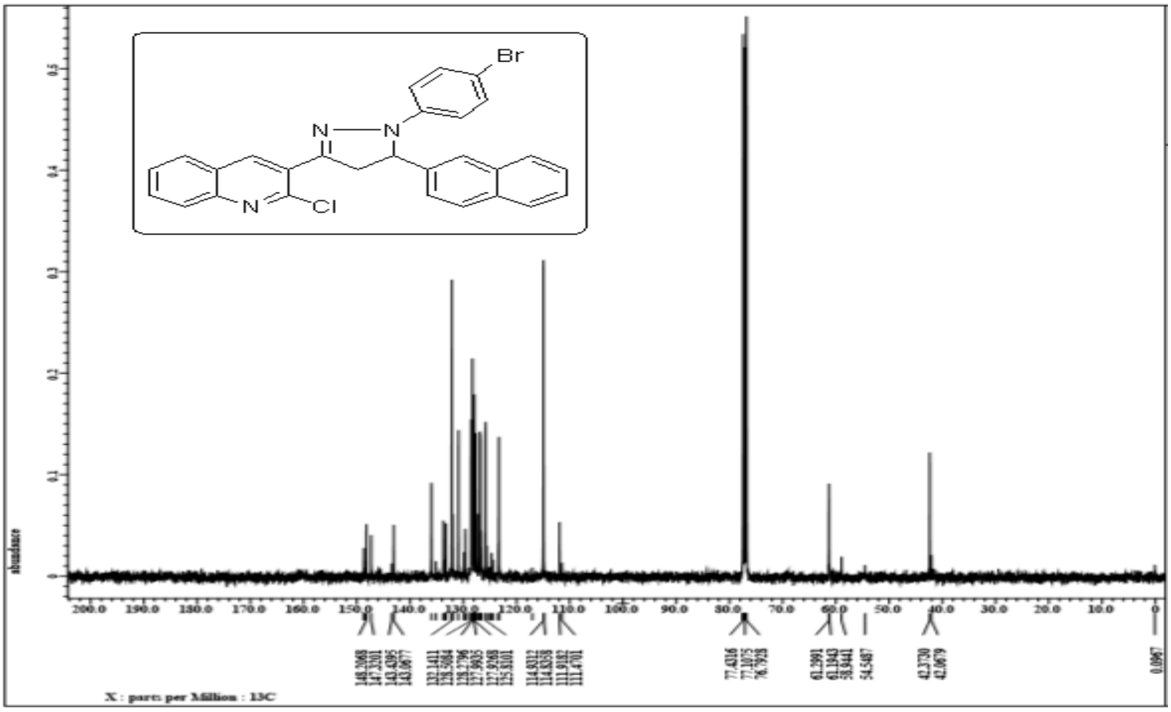

^{13}C NMR spectrum of compound 9

Compound 10:- 3-(5-(4-Bromophenyl)-1-(3-fluorophenyl)-4,5-dihydro-1H-pyrazol-3-yl)-2-chloroquinoline

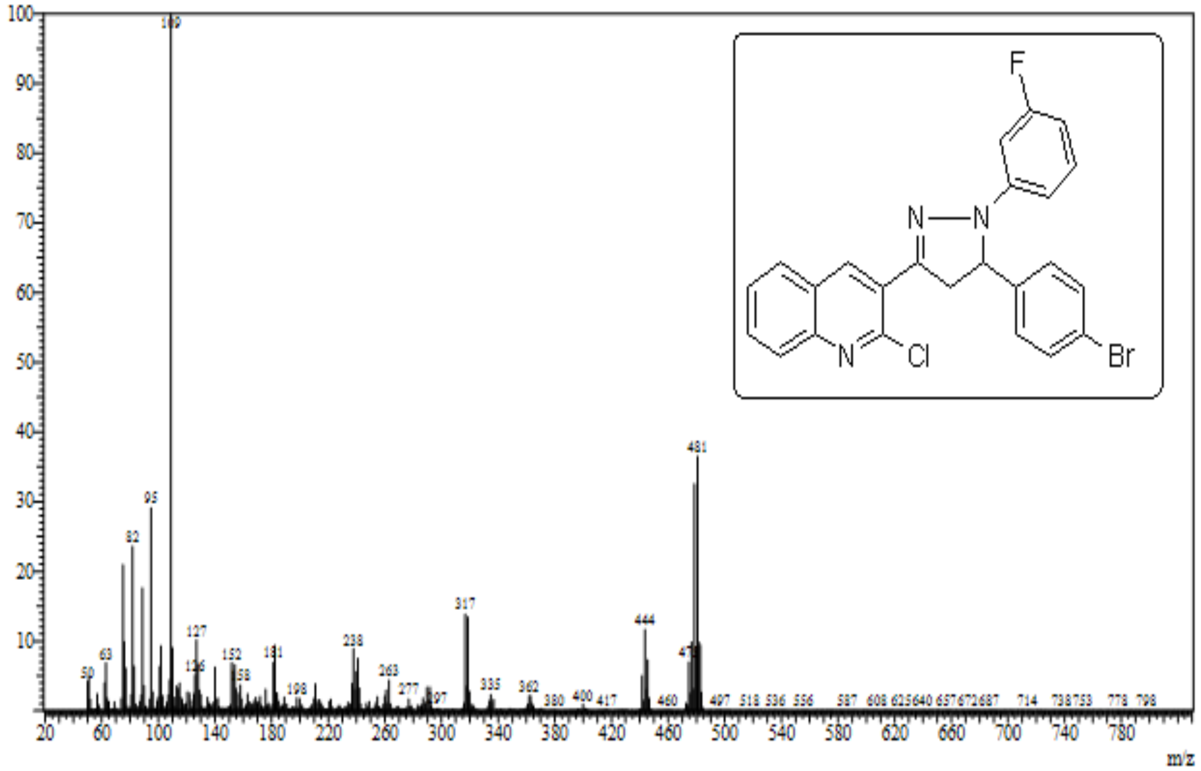

Mass spectrum of compound 10

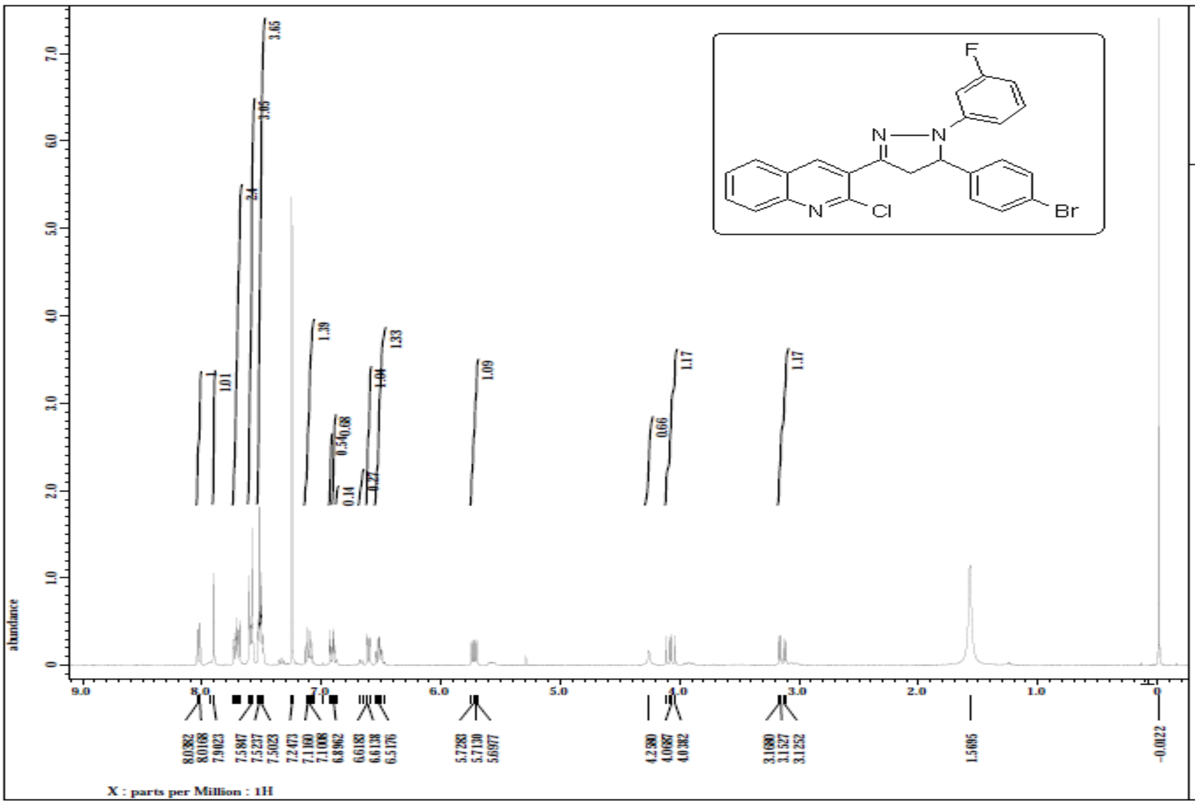

Proton NMR of Compound 10

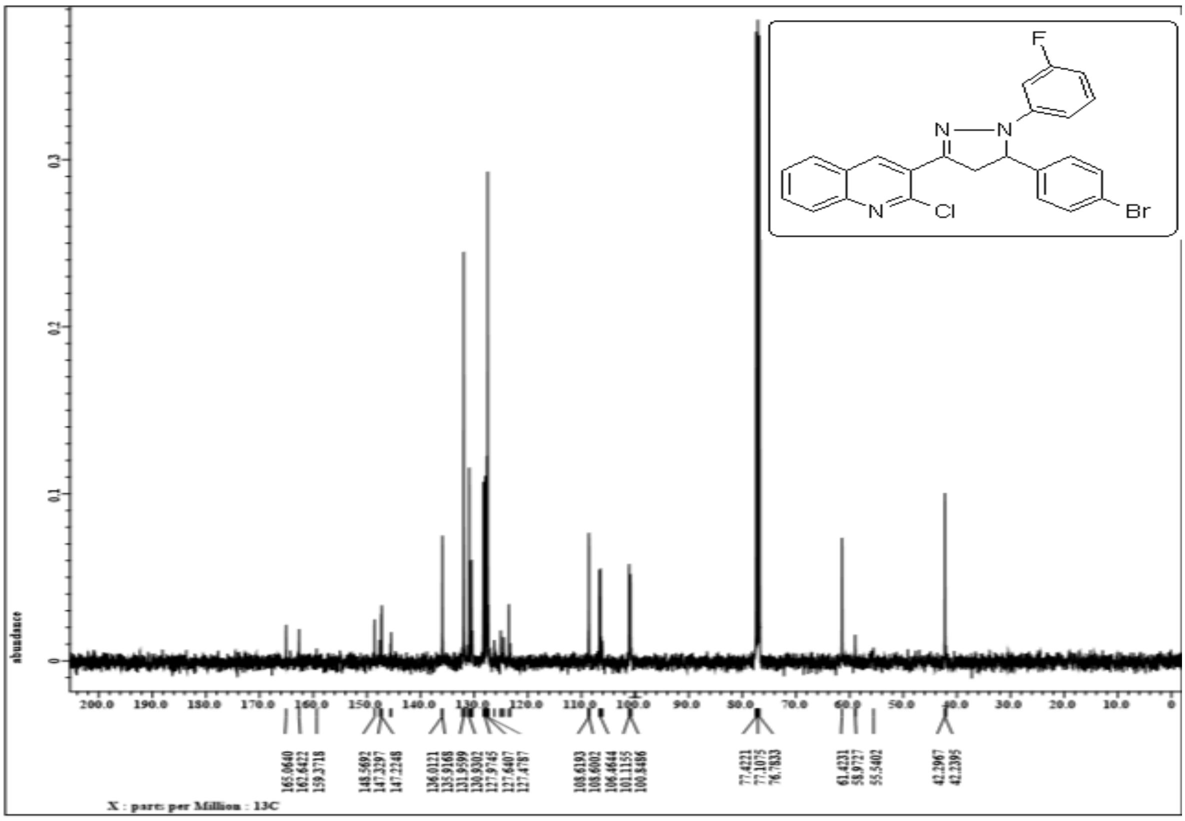

^{13}C NMR spectrum of Compound 10

Compound 11:- 3-(1-(3-Bromophenyl)-5-(4-bromophenyl)-4,5-dihydro-1H-pyrazol-3-yl)-2-chloroquinoline

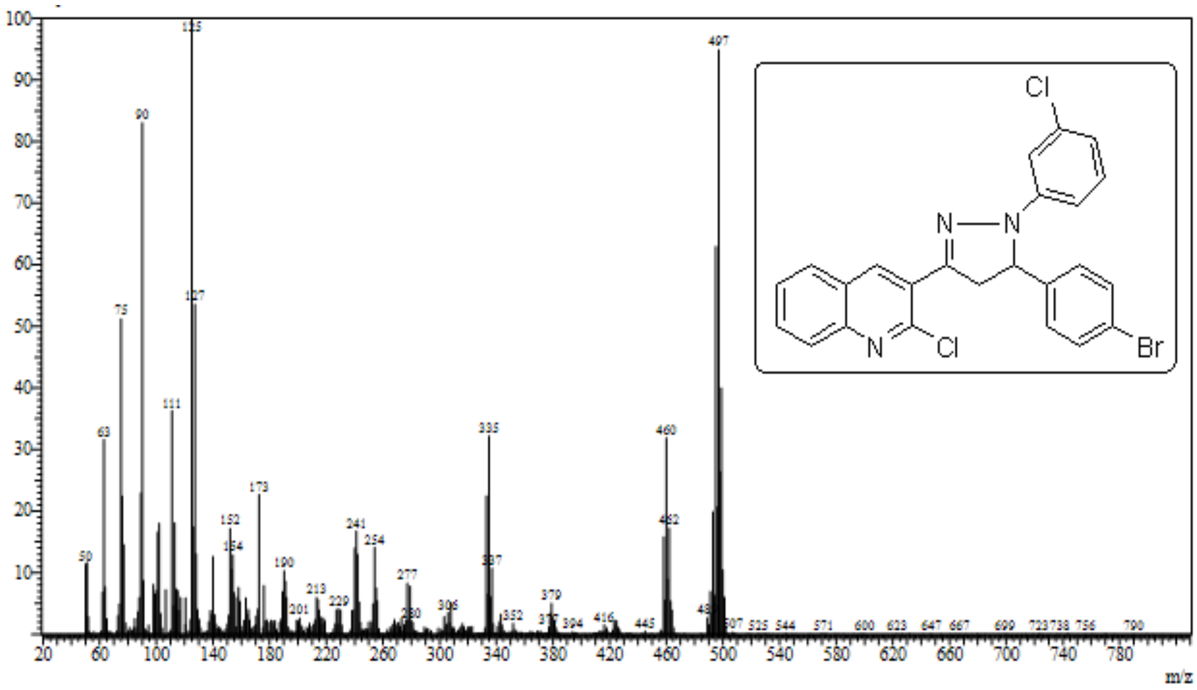

Mass spectrum of compound 11

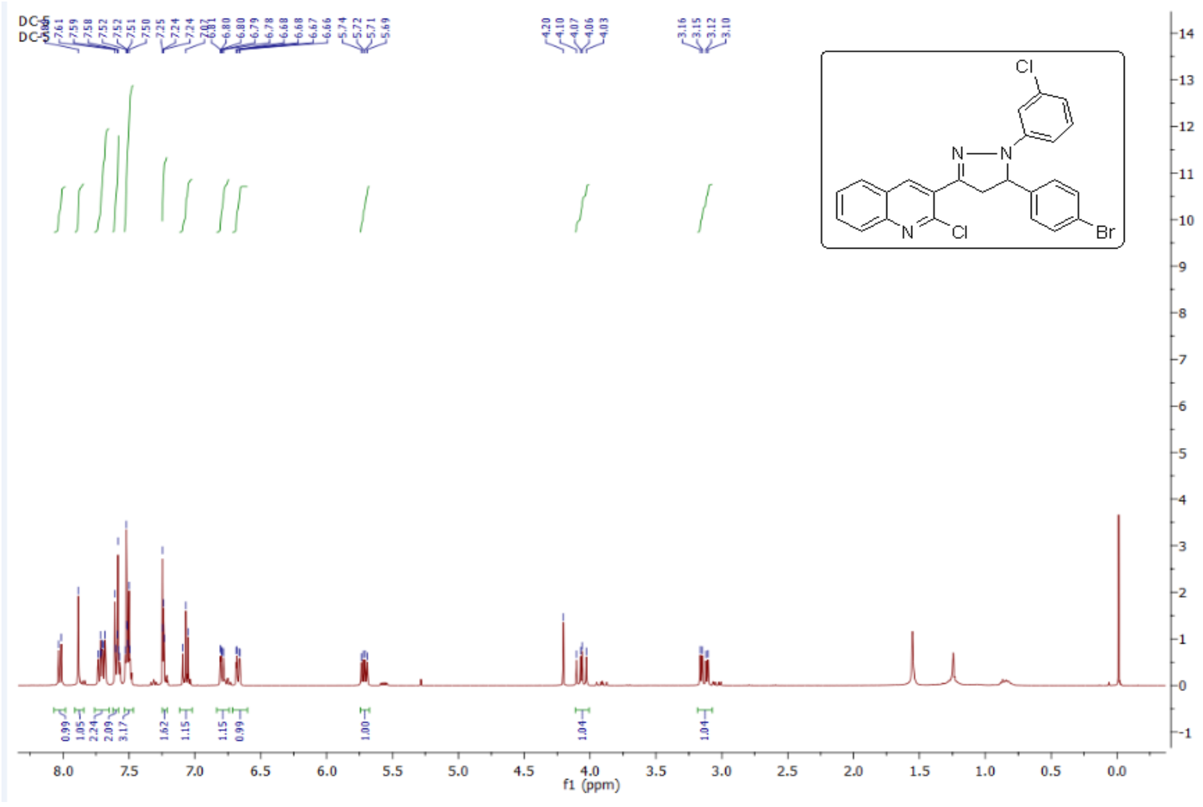

Proton NMR spectrum of Compound 11

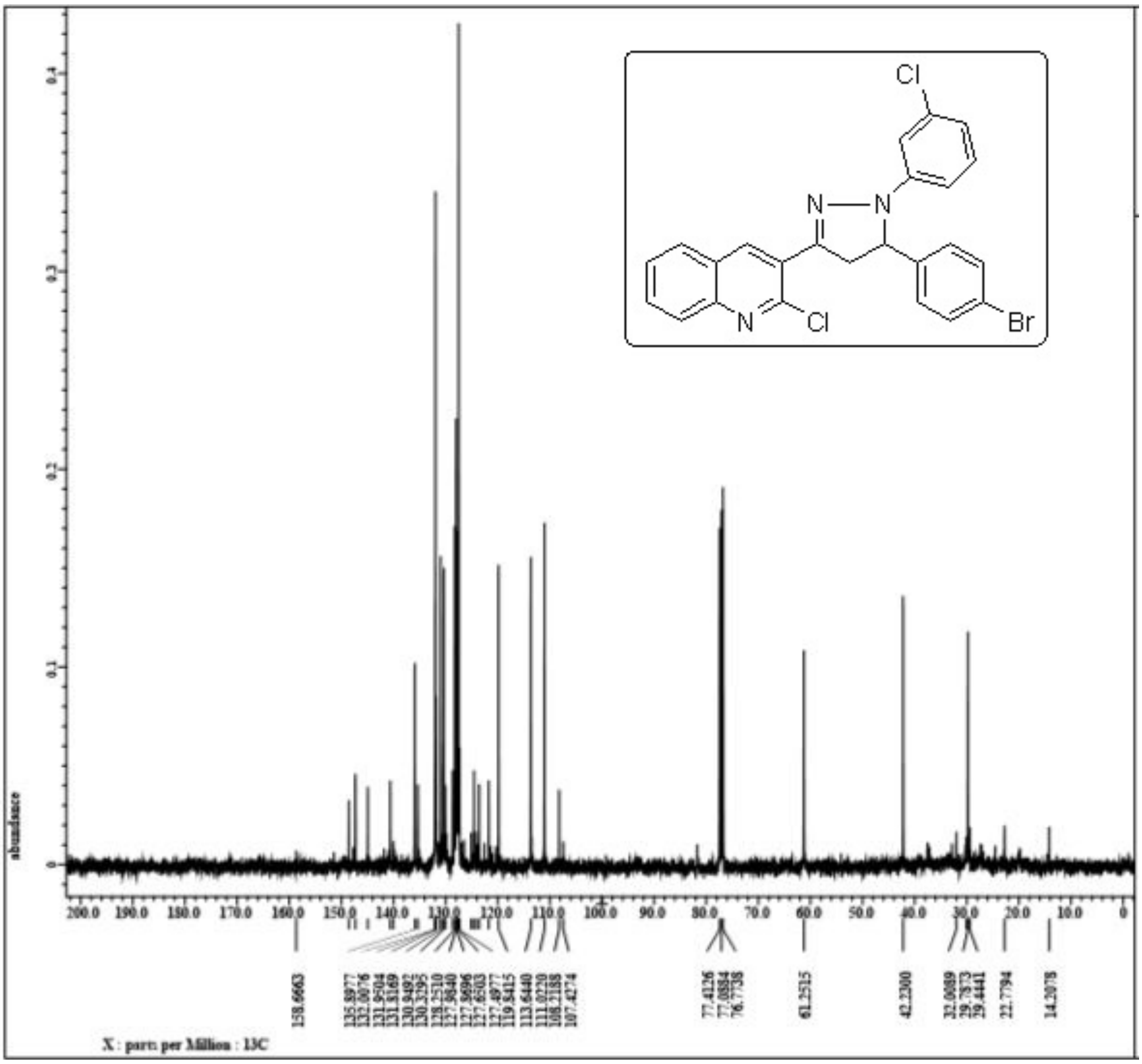

13C-NMR of Compound 11

Compound 12:- 5-(4-Bromophenyl)-3-(3-chloronaphthalen-2-yl)-1-(4-fluorophenyl)-4,5-dihydro-1H-pyrazole

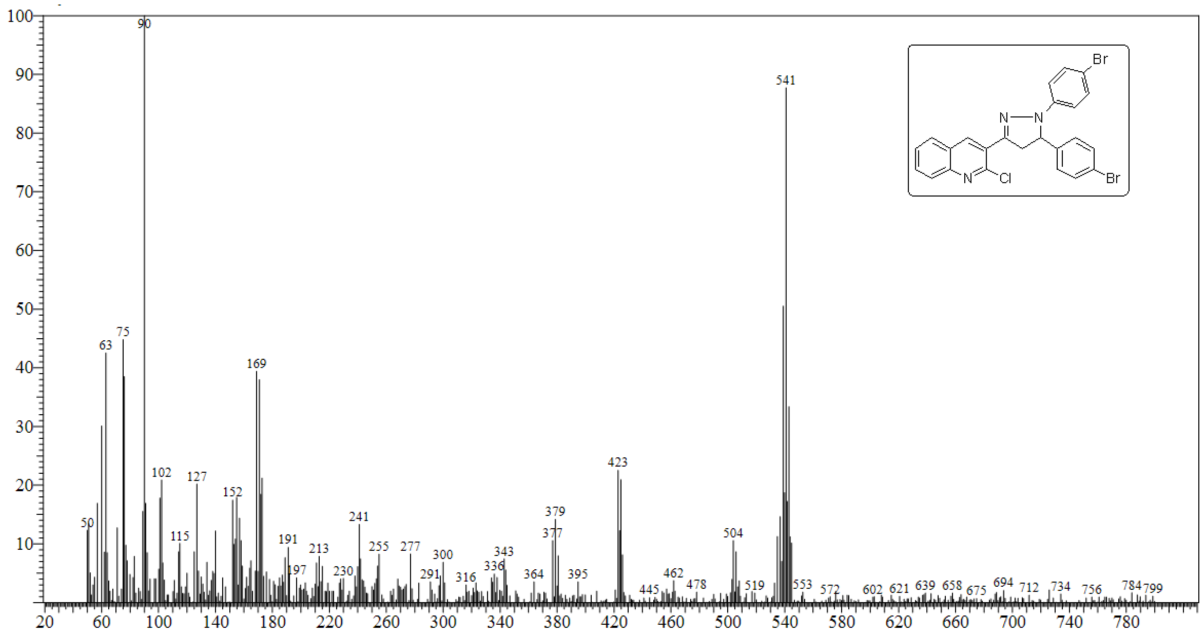

Mass spectrum of compound 12

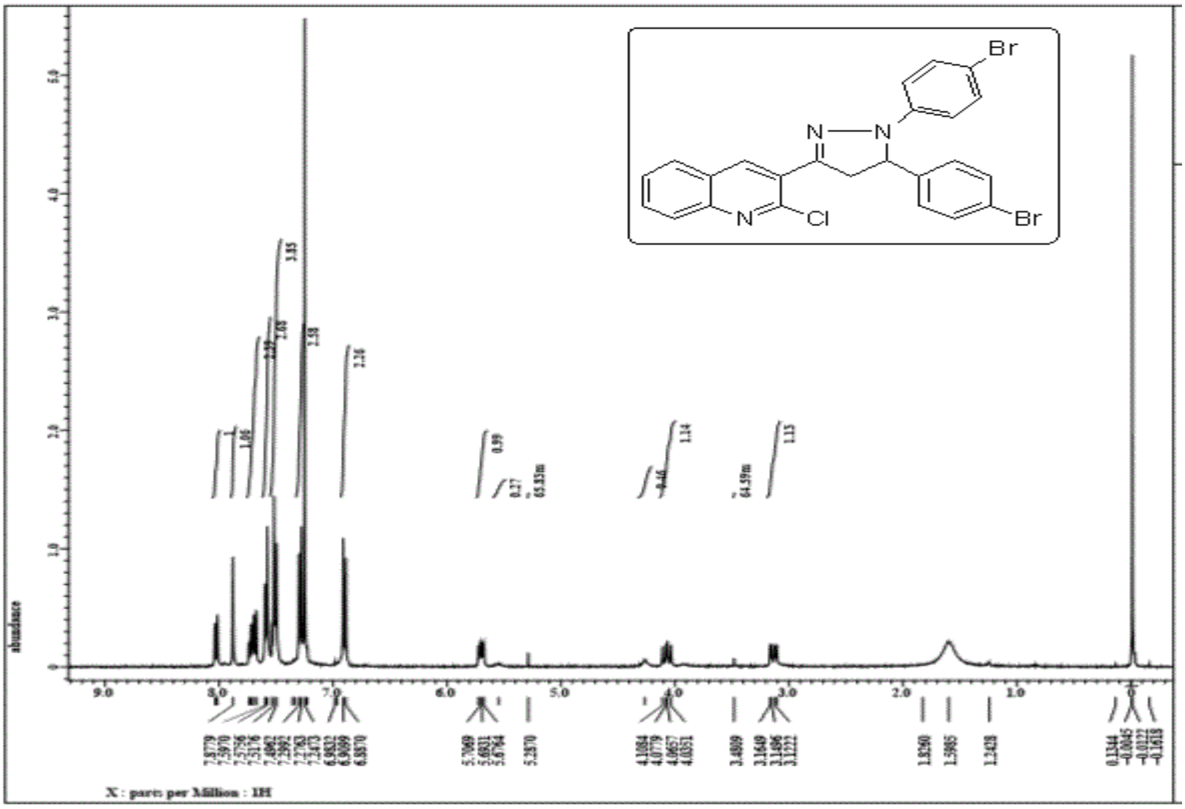

Proton NMR of compound 12

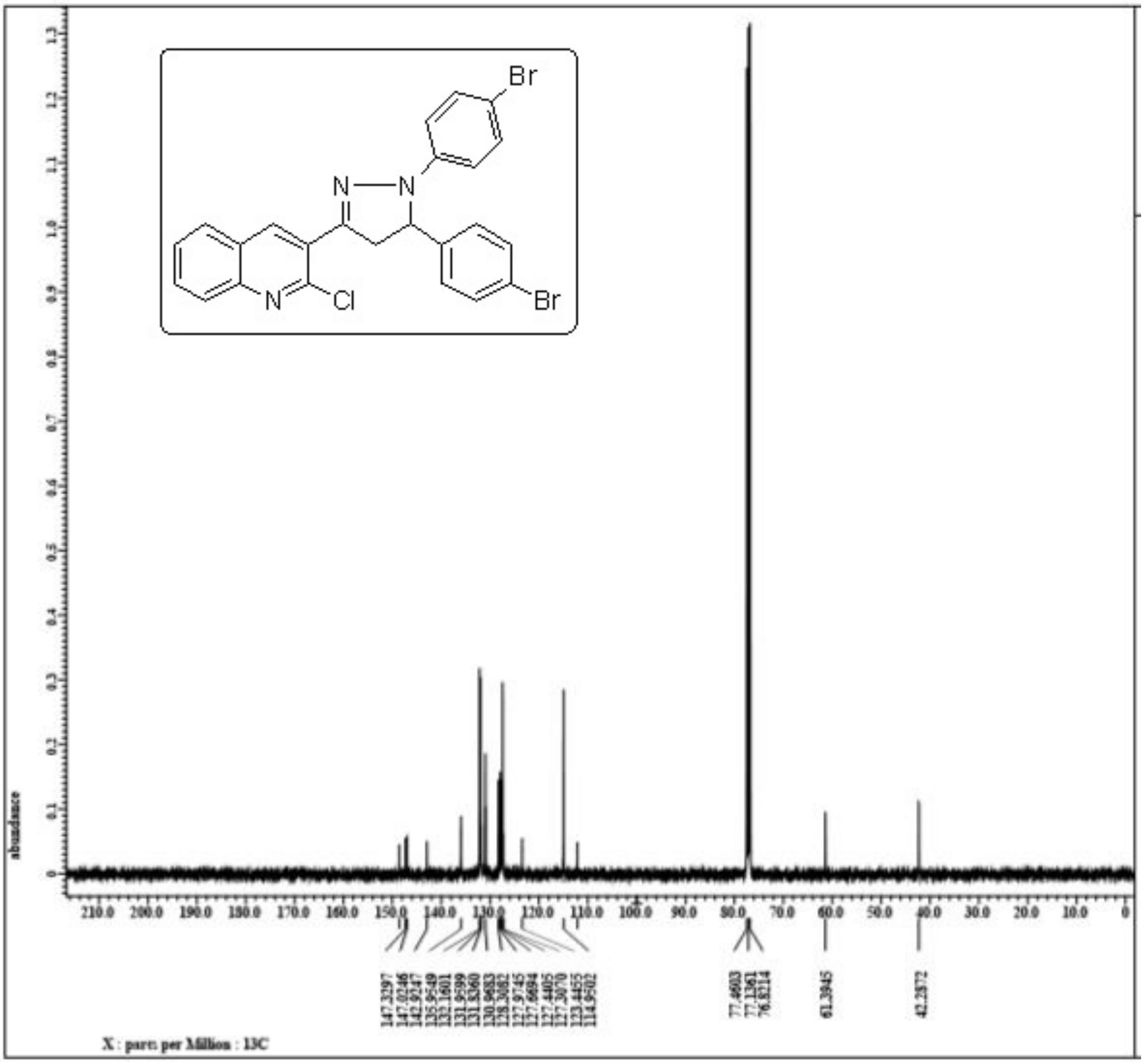

^{13}C-NMR spectrum of compound 12

Compound 13:- 3-(1,5-Bis(4-bromophenyl)-4,5-dihydro-1H-pyrazol-3-yl)-2-chloroquinoline

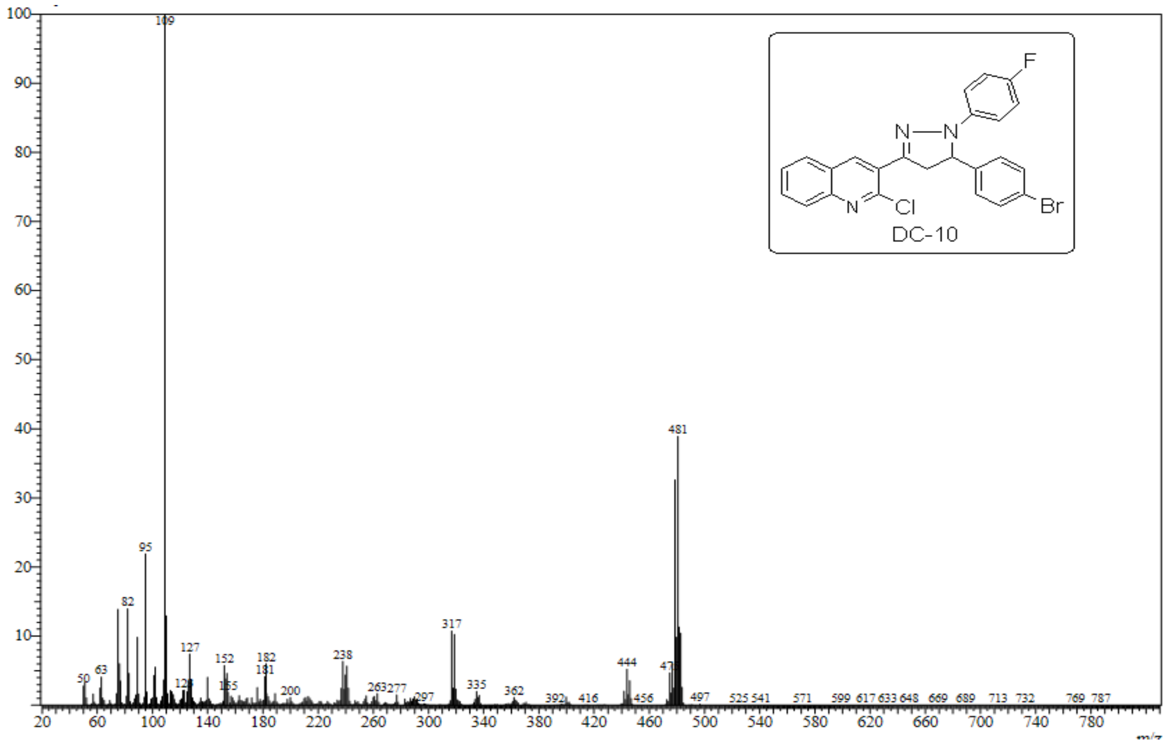

Mass spectrum of compound 13

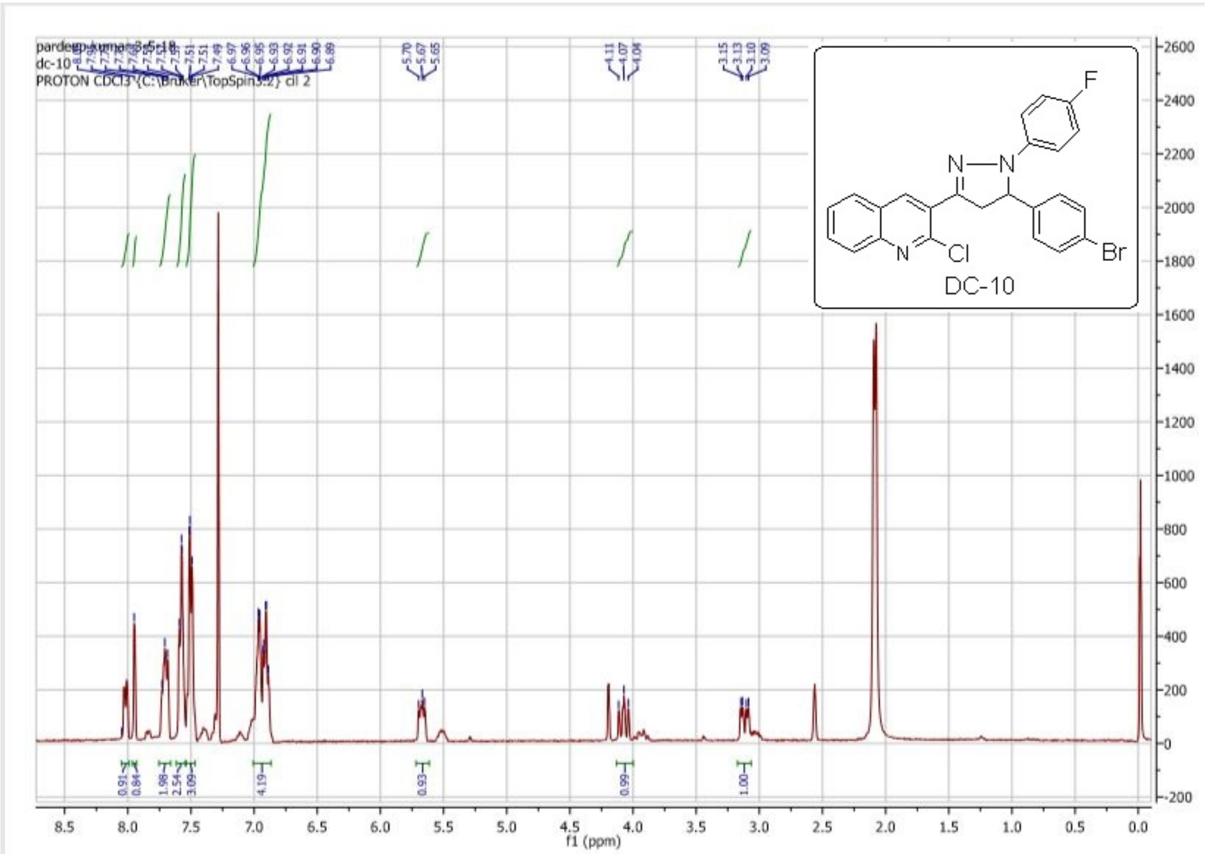

Proton NMR of Compound 13

Compound 14 :- 3-(1-(4-Bromophenyl)-5-(naphthalen-2-yl)-4,5-dihydro-1H-pyrazol-3-yl)-2-chloroquinoline

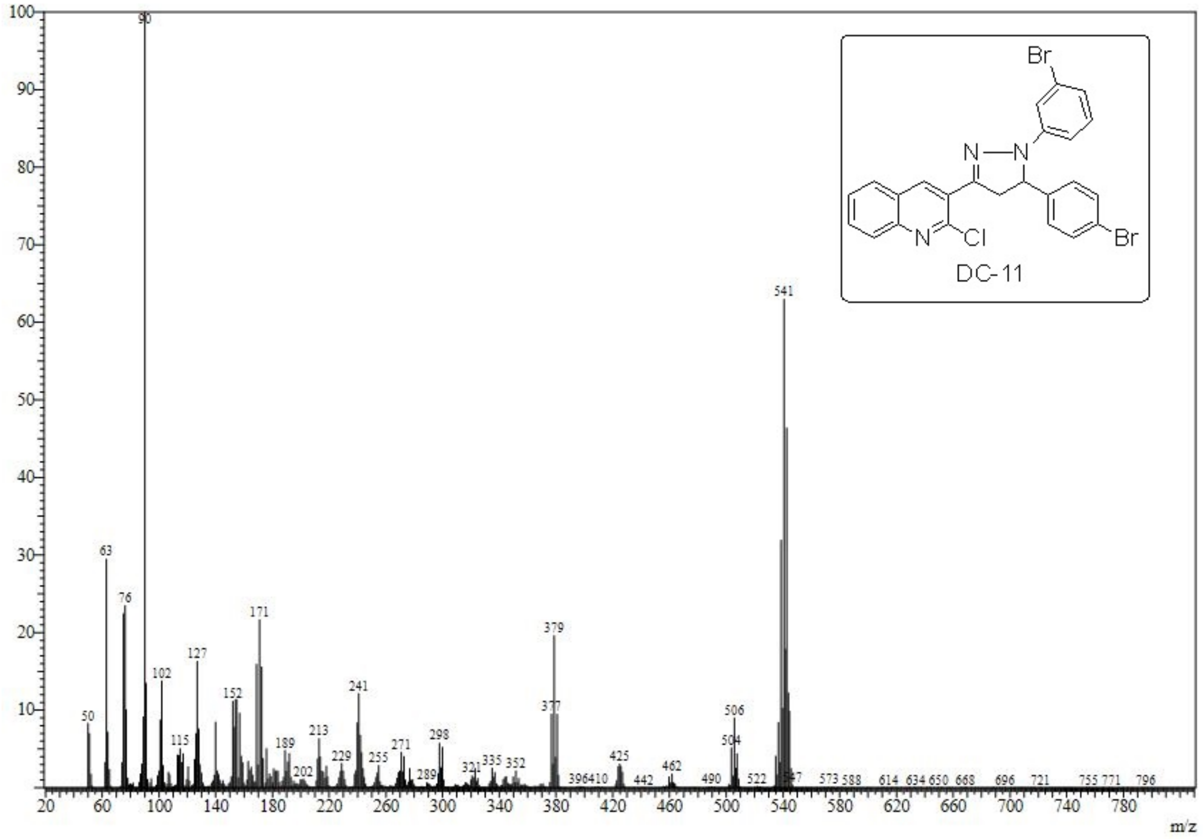

Mass spectrum of compound 14

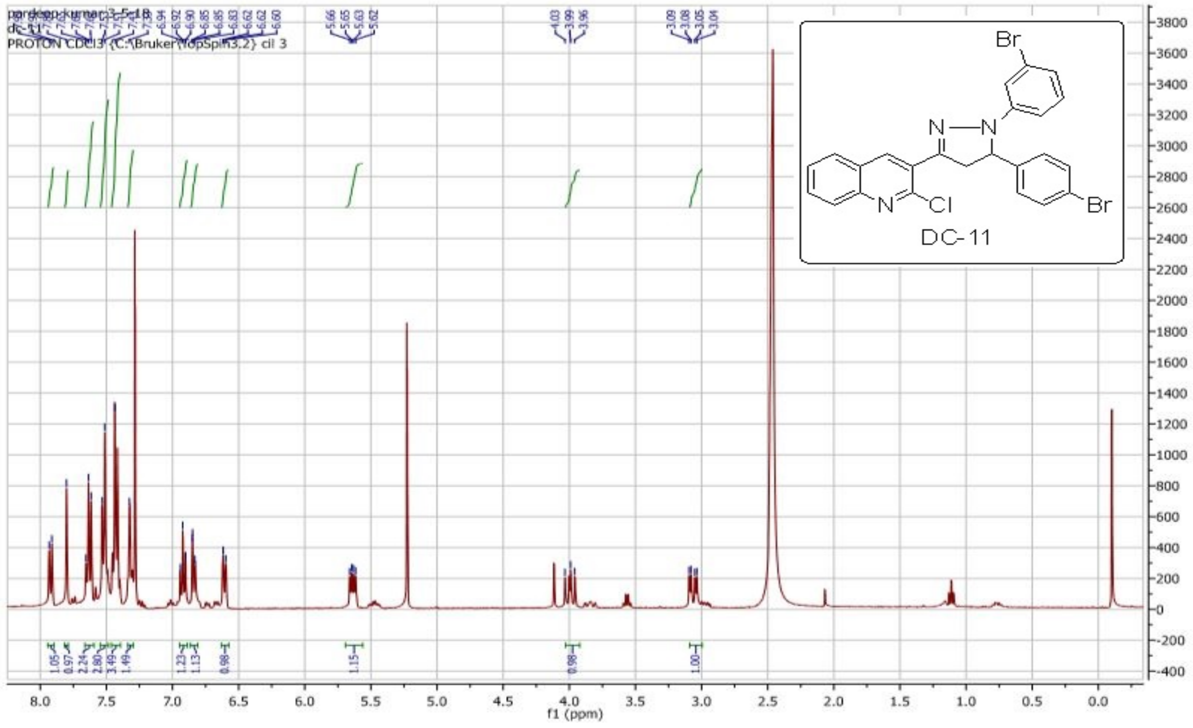

Proton NMR spectrum of compound 14

SciFinder search results

Compound 4 – Zero results

Compound 5- Zero results

Compound 6-Zero results

Compound 7-Zero results

Compound 8-Zero results

Compound 9-Zero results

Compound 10-Zero results

Compound 11-Zero results

Compound 13-Zero results

Compound 12-Zero results

Compound 14-Zero results

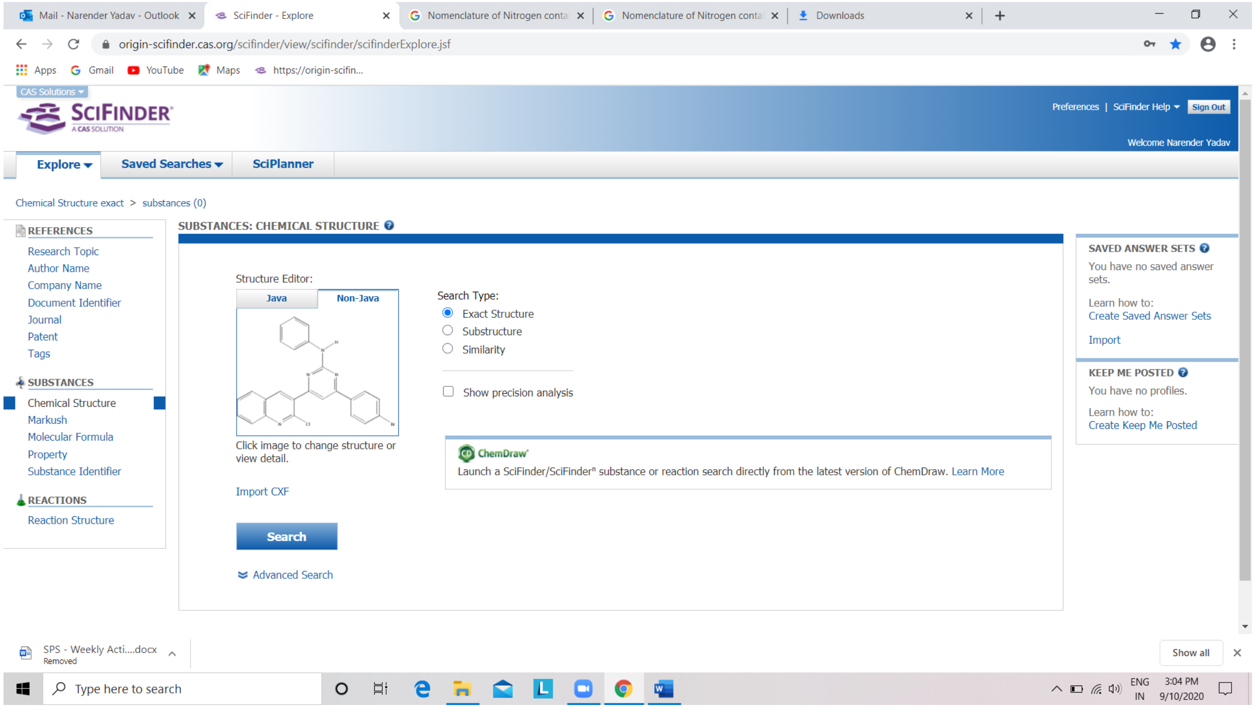

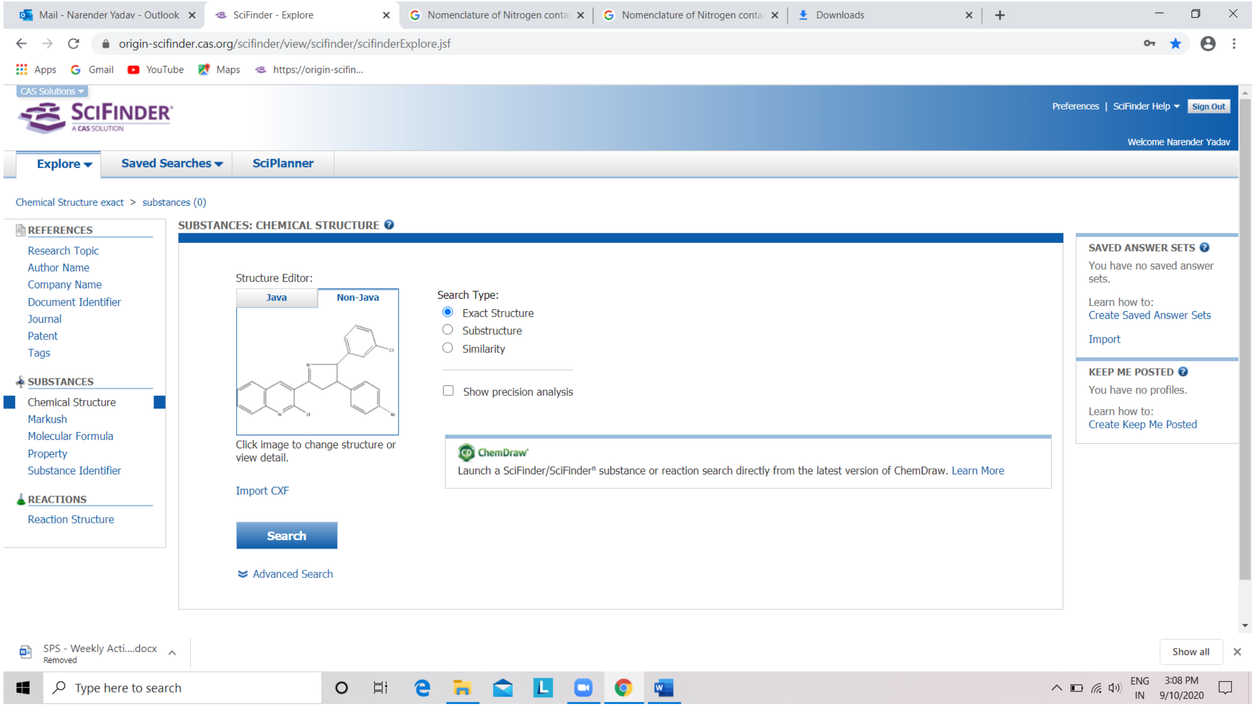

Mail - Narender Yadav - Outlook
SciFinder - Explore
Nomenclature of Nitrogen conta
Downloads
origin-scifinder.cas.org/scifinder/view/scifinder/scifinderExplore.jsf
Apps
Gmail
YouTube
Maps
https://origin-scifin...
CAS Solutions
SCIFINDER
A CAS SOLUTION
Preferences | SciFinder Help
Sign Out
Welcome Narender Yadav
Explore
Saved Searches
SciPlanner
Chemical Structure exact > substances (0)
REFERENCES
Research Topic
Author Name
Company Name
Document Identifier
Journal
Patent
Tags
SUBSTANCES
Chemical Structure
Markush
Molecular Formula
Property
Substance Identifier
REACTIONS
Reaction Structure
SUBSTANCES: CHEMICAL STRUCTURE
Structure Editor:
Java
Non-Java
Click image to change structure or view detail.
Import CXF
Search
Advanced Search
Search Type:
Exact Structure
Substructure
Similarity
Show precision analysis
ChemDraw
Launch a SciFinder/SciFinder® substance or reaction search directly from the latest version of ChemDraw. Learn More
SAVED ANSWER SETS
You have no saved answer sets.
Learn how to:
Create Saved Answer Sets
Import
KEEP ME POSTED
You have no profiles.
Learn how to:
Create Keep Me Posted
SPS - Weekly Acti....docx
Removed
Show all
Type here to search
ENG
IN
3:08 PM
9/10/2020

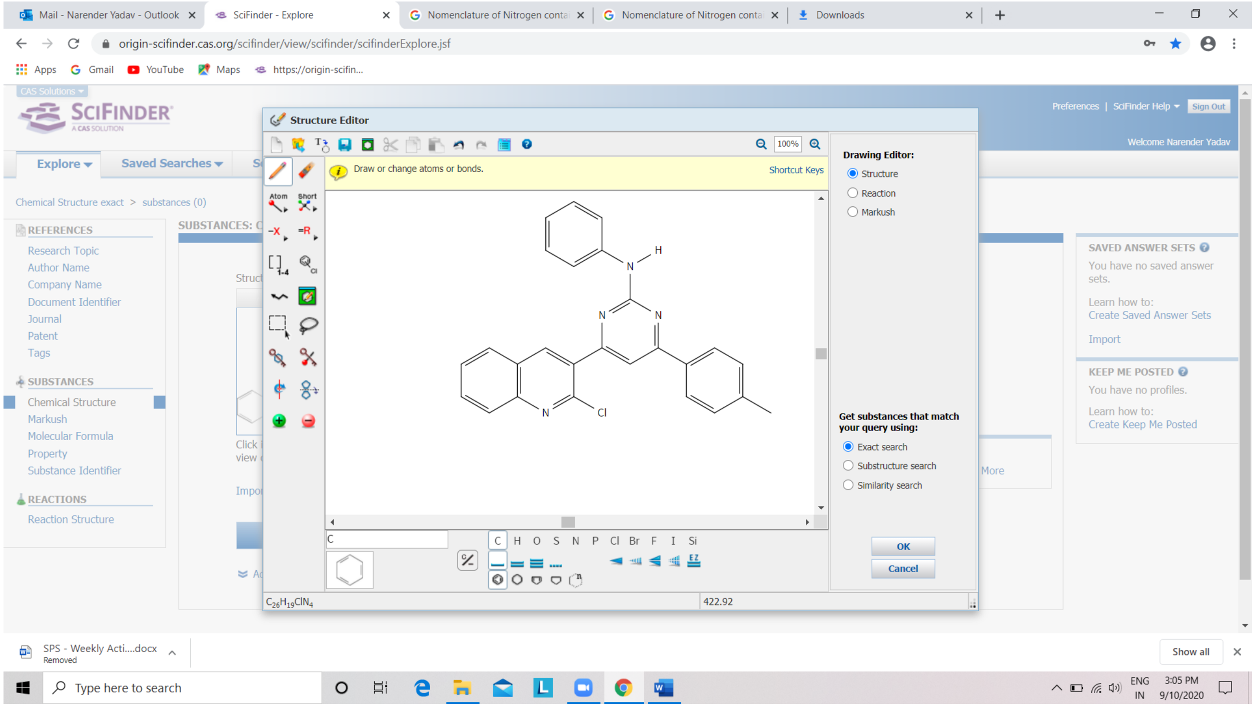

Mail - Narender Yadav - Outlook
SciFinder - Explore
Nomenclature of Nitrogen conta
Downloads
origin-scifinder.cas.org/scifinder/view/scifinder/scifinderExplore.jsf
Structure Editor
100%
Draw or change atoms or bonds.
Shortcut Keys
Atom
Short
Drawing Editor:
Structure
Reaction
Markush
N
H
N
N
N
Cl
Get substances that match your query using:
Exact search
Substructure search
Similarity search
OK
Cancel
C H O S N P Cl Br F I Si
C26H19ClN4
422.92
SAVED ANSWER SETS
You have no saved answer sets.
KEEP ME POSTED
You have no profiles.
SPS - Weekly Acti....docx
Removed
Show all
Type here to search
ENG
IN
3:05 PM
9/10/2020

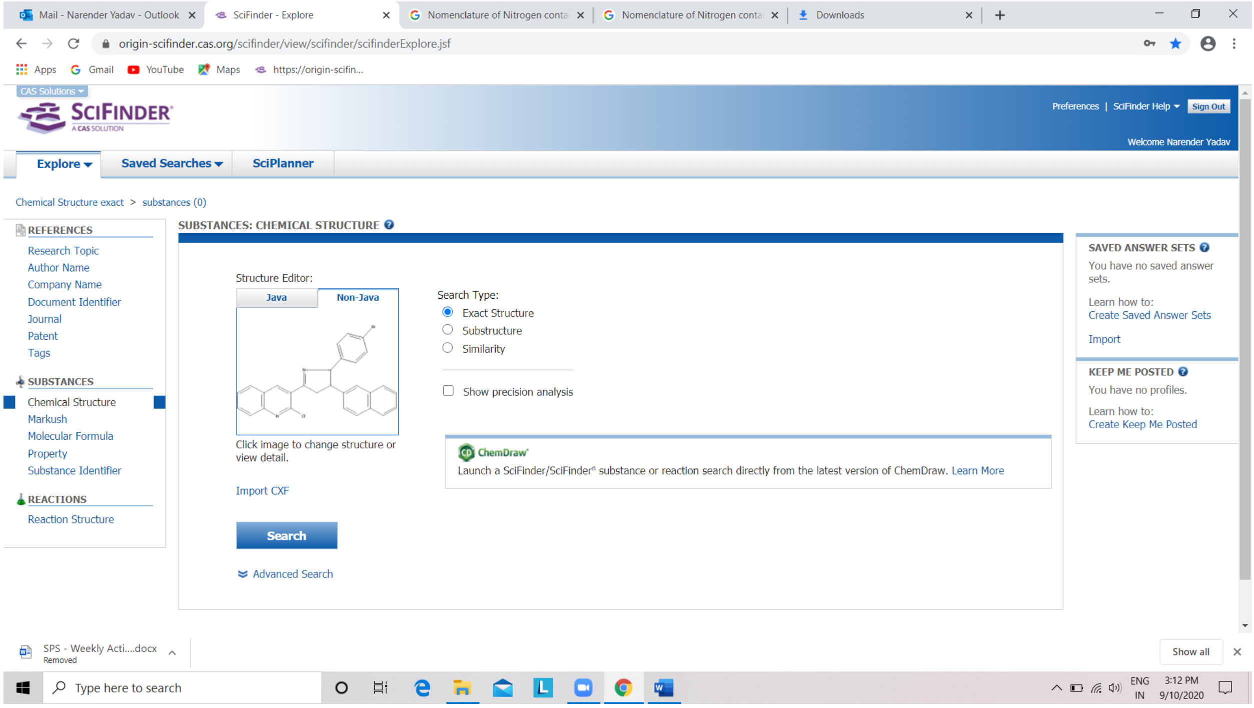
Mail - Narender Yadav - Outlook
SciFinder - Explore
Nomenclature of Nitrogen conta
Nomenclature of Nitrogen conta
Downloads
origin-scifinder.cas.org/scifinder/view/scifinder/scifinderExplore.jsf
Apps Gmail YouTube Maps https://origin-scifin...
CAS Solutions
SCIFINDER
A CAS SOLUTION
Preferences | SciFinder Help | Sign Out
Welcome Narender Yadav
Explore
Saved Searches
SciPlanner
Chemical Structure exact > substances (0)
REFERENCES
Research Topic
Author Name
Company Name
Document Identifier
Journal
Patent
Tags
SUBSTANCES
Chemical Structure
Markush
Molecular Formula
Property
Substance Identifier
REACTIONS
Reaction Structure
SUBSTANCES: CHEMICAL STRUCTURE
Structure Editor:
Java
Non-Java
Click image to change structure or view detail.
Import CXF
Search
Advanced Search
Search Type:
Exact Structure
Substructure
Similarity
Show precision analysis
ChemDraw
Launch a SciFinder/SciFinder® substance or reaction search directly from the latest version of ChemDraw. Learn More
SAVED ANSWER SETS
You have no saved answer sets.
Learn how to:
Create Saved Answer Sets
Import
KEEP ME POSTED
You have no profiles.
Learn how to:
Create Keep Me Posted
SPS - Weekly Acti....docx
Removed
Show all
Type here to search
ENG
IN
3:12 PM
9/10/2020

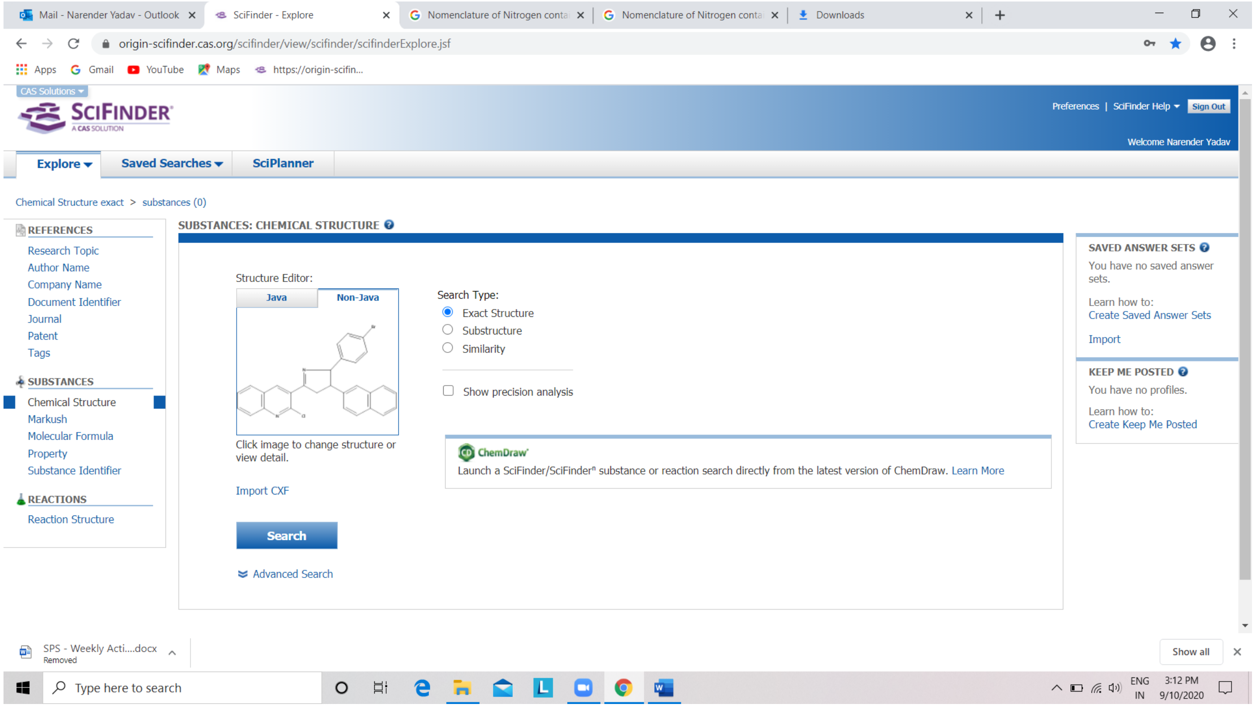
Mail - Narender Yadav - Outlook
SciFinder - Explore
Nomenclature of Nitrogen conta
Nomenclature of Nitrogen conta
Downloads
origin-scifinder.cas.org/scifinder/view/scifinder/scifinderExplore.jsf
Apps Gmail YouTube Maps https://origin-scifin...
CAS Solutions
SCIFINDER
A CAS SOLUTION
Preferences | SciFinder Help | Sign Out
Welcome Narender Yadav
Explore
Saved Searches
SciPlanner
Chemical Structure exact > substances (0)
REFERENCES
Research Topic
Author Name
Company Name
Document Identifier
Journal
Patent
Tags
SUBSTANCES
Chemical Structure
Markush
Molecular Formula
Property
Substance Identifier
REACTIONS
Reaction Structure
SUBSTANCES: CHEMICAL STRUCTURE
Structure Editor:
Java
Non-Java
Click image to change structure or view detail.
Import CXF
Search
Advanced Search
Search Type:
Exact Structure
Substructure
Similarity
Show precision analysis
ChemDraw
Launch a SciFinder/SciFinder® substance or reaction search directly from the latest version of ChemDraw. Learn More
SAVED ANSWER SETS
You have no saved answer sets.
Learn how to:
Create Saved Answer Sets
Import
KEEP ME POSTED
You have no profiles.
Learn how to:
Create Keep Me Posted
SPS - Weekly Acti....docx
Removed
Show all
Type here to search
ENG
IN
3:12 PM
9/10/2020

Mail - Narender Yadav - Outlook | SciFinder - Substance Answer Set

origin-scifinder.cas.org/scifinder/view/scifinder/scifinderExplore.jsf

Apps Gmail YouTube Maps https://origin-scifin...

CAS Solutions

SCIFINDER A CAS SOLUTION

Preferences | SciFinder Help | Sign Out

Welcome Narender Yadav

Explore | Saved Searches | SciPlanner

Explore Substances resulted in 0 substances Return

Chemical Structure exact > **substances (0)**

Create Keep Me Posted Alert

Explore substances by structure: substances *exact*

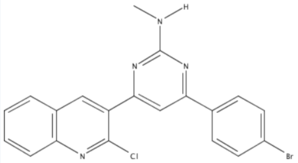

Resulting in 0 candidate

Contact Us | Legal

Copyright © 2020 American Chemical Society. All Rights Reserved. | 京ICP备13047075号-3

SPS - Weekly Acti....docx
Removed

Show all

Type here to search

ENG IN 4:41 PM 9/10/2020

Mail - Narender Yadav - Outlook | SciFinder - Substance Answer Set

origin-scifinder.cas.org/scifinder/view/scifinder/scifinderExplore.jsf

Apps Gmail YouTube Maps https://origin-scifin...

CAS Solutions

SCIFINDER A CAS SOLUTION

Preferences | SciFinder Help | Sign Out

Welcome Narender Yadav

Explore | Saved Searches | SciPlanner

Explore Substances resulted in 0 substances Return

Chemical Structure exact > **substances (0)**

Create Keep Me Posted Alert

Explore substances by structure: substances *exact*

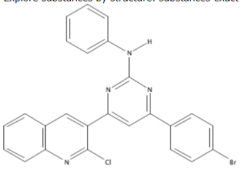

Resulting in 0 candidate

Contact Us | Legal

Copyright © 2020 American Chemical Society. All Rights Reserved. | 京ICP备13047075号-3

SPS - Weekly Acti....docx
Removed

Show all

Type here to search

ENG IN 4:41 PM 9/10/2020

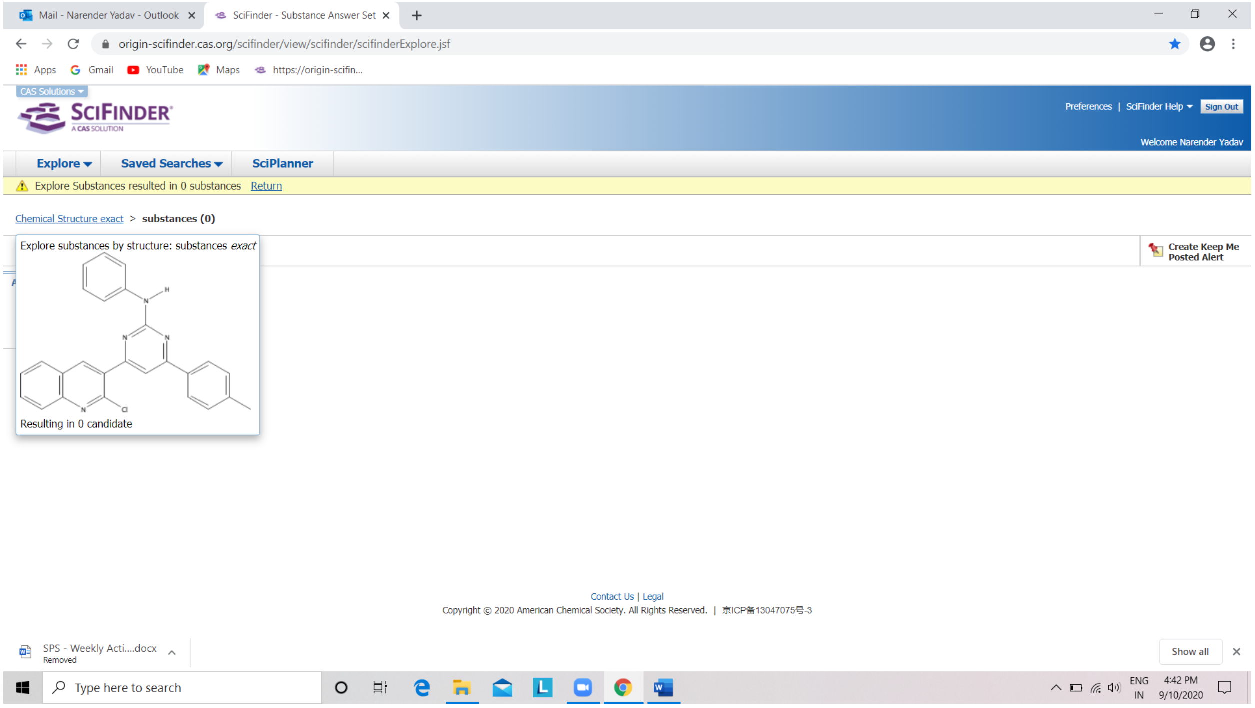
Mail - Narender Yadav - Outlook
SciFinder - Substance Answer Set
origin-scifinder.cas.org/scifinder/view/scifinder/scifinderExplore.jsf
Apps Gmail YouTube Maps https://origin-scifin...
CAS Solutions
SCIFINDER
A CAS SOLUTION
Preferences | SciFinder Help Sign Out
Welcome Narender Yadav
Explore Saved Searches SciPlanner
Explore Substances resulted in 0 substances Return
Chemical Structure exact > substances (0)
Explore substances by structure: substances exact
Create Keep Me Posted Alert
Resulting in 0 candidate
Contact Us | Legal
Copyright © 2020 American Chemical Society. All Rights Reserved. | 京ICP备13047075号-3
SPS - Weekly Acti....docx
Removed
Show all
Type here to search
ENG IN 4:42 PM 9/10/2020

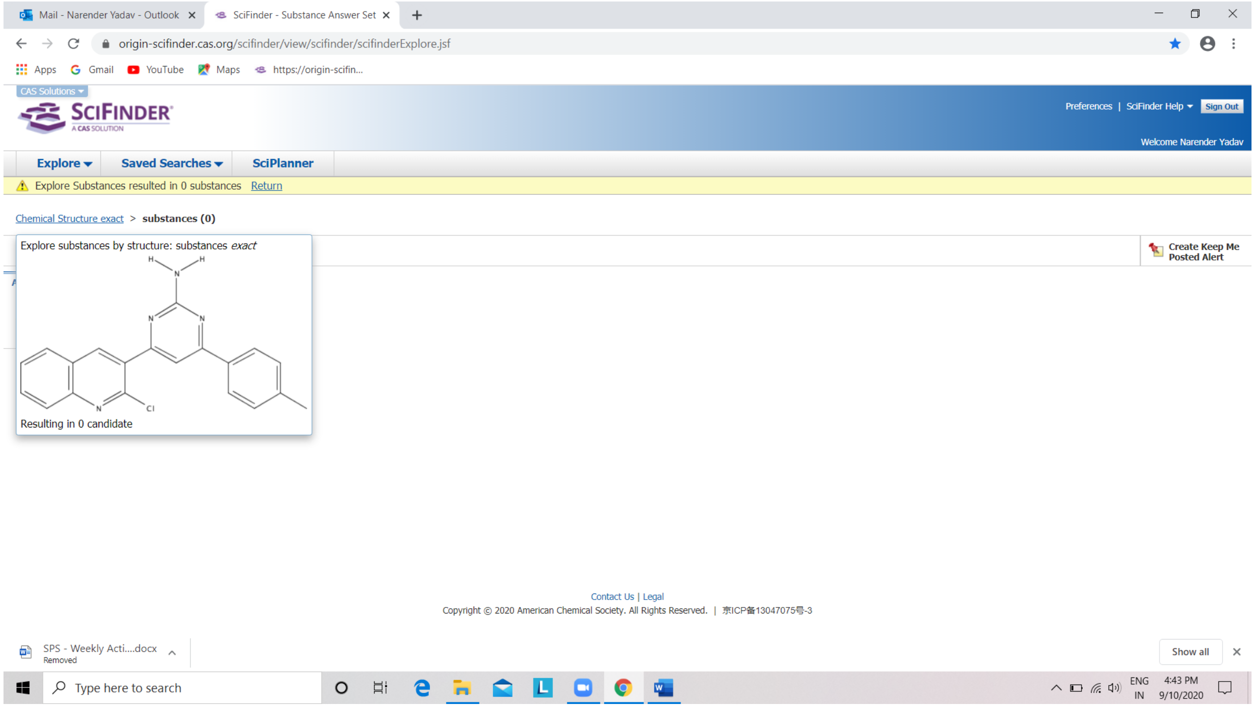
Mail - Narender Yadav - Outlook
SciFinder - Substance Answer Set
origin-scifinder.cas.org/scifinder/view/scifinder/scifinderExplore.jsf
Apps Gmail YouTube Maps https://origin-scifin...
CAS Solutions
SCIFINDER
A CAS SOLUTION
Preferences | SciFinder Help Sign Out
Welcome Narender Yadav
Explore Saved Searches SciPlanner
Explore Substances resulted in 0 substances Return
Chemical Structure exact > substances (0)
Explore substances by structure: substances exact
Create Keep Me Posted Alert
Resulting in 0 candidate
Contact Us | Legal
Copyright © 2020 American Chemical Society. All Rights Reserved. | 京ICP备13047075号-3
SPS - Weekly Acti....docx
Removed
Show all
Type here to search
ENG IN 4:43 PM 9/10/2020

Mail - Narender Yadav - Outlook | SciFinder - Substance Answer Set

origin-scifinder.cas.org/scifinder/view/scifinder/scifinderExplore.jsf

Apps | Gmail | YouTube | Maps | https://origin-scifin...

CAS Solutions

SCIFINDER A CAS SOLUTION

Preferences | SciFinder Help | Sign Out

Welcome Narender Yadav

Explore | Saved Searches | SciPlanner

Explore Substances resulted in 0 substances Return

Chemical Structure exact > **substances (0)**

Explore substances by structure: substances *exact*

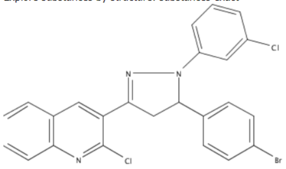

Resulting in 0 candidate

Create Keep Me Posted Alert

Contact Us | Legal

Copyright © 2020 American Chemical Society. All Rights Reserved. | 京ICP备13047075号-3

SPS - Weekly Acti....docx
Removed

Show all

Type here to search

ENG IN 4:45 PM 9/10/2020

Mail - Narender Yadav - Outlook | SciFinder - Substance Answer Set

origin-scifinder.cas.org/scifinder/view/scifinder/scifinderExplore.jsf

Apps | Gmail | YouTube | Maps | https://origin-scifin...

CAS Solutions

SCIFINDER A CAS SOLUTION

Preferences | SciFinder Help | Sign Out

Welcome Narender Yadav

Explore | Saved Searches | SciPlanner

Explore Substances resulted in 0 substances Return

Chemical Structure exact > **substances (0)**

Explore substances by structure: substances *exact*

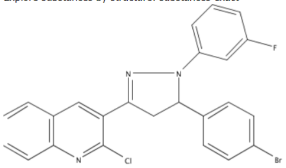

Resulting in 0 candidate

Create Keep Me Posted Alert

Contact Us | Legal

Copyright © 2020 American Chemical Society. All Rights Reserved. | 京ICP备13047075号-3

SPS - Weekly Acti....docx
Removed

Show all

Type here to search

ENG IN 4:45 PM 9/10/2020

Mail - Narender Yadav - Outlook | SciFinder - Substance Answer Set

origin-scifinder.cas.org/scifinder/view/scifinder/scifinderExplore.jsf

Apps | Gmail | YouTube | Maps | https://origin-scifin...

CAS Solutions

SCIFINDER A CAS SOLUTION

Preferences | SciFinder Help | Sign Out

Welcome Narender Yadav

Explore | Saved Searches | SciPlanner

Explore Substances resulted in 0 substances Return

Chemical Structure exact > **substances (0)**

Explore substances by structure: substances *exact*

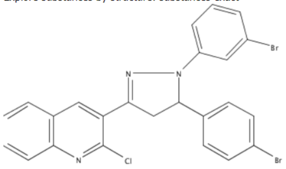

Resulting in 0 candidate

Create Keep Me Posted Alert

Contact Us | Legal

Copyright © 2020 American Chemical Society. All Rights Reserved. | 京ICP备13047075号-3

SPS - Weekly Acti....docx
Removed

Show all

Type here to search

ENG IN 4:46 PM 9/10/2020

Mail - Narender Yadav - Outlook | SciFinder - Substance Answer Set

origin-scifinder.cas.org/scifinder/view/scifinder/scifinderExplore.jsf

Apps | Gmail | YouTube | Maps | https://origin-scifin...

CAS Solutions

SCIFINDER A CAS SOLUTION

Preferences | SciFinder Help | Sign Out

Welcome Narender Yadav

Explore | Saved Searches | SciPlanner

Explore Substances resulted in 0 substances Return

Chemical Structure exact > **substances (0)**

Explore substances by structure: substances *exact*

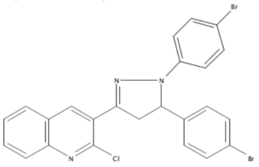

Resulting in 0 candidate

Create Keep Me Posted Alert

Contact Us | Legal

Copyright © 2020 American Chemical Society. All Rights Reserved. | 京ICP备13047075号-3

SPS - Weekly Acti....docx
Removed

Show all

Type here to search

ENG IN 4:47 PM 9/10/2020

Mail - Narender Yadav - Outlook × SciFinder - Substance Answer Set × +

origin-scifinder.cas.org/scifinder/view/scifinder/scifinderExplore.jsf

Apps Gmail YouTube Maps https://origin-scifin...

CAS Solutions

SCIFINDER A CAS SOLUTION

Preferences | SciFinder Help Sign Out

Welcome Narender Yadav

Explore Saved Searches SciPlanner

Explore Substances resulted in 0 substances Return

Chemical Structure exact > **substances (0)**

Explore substances by structure: substances *exact*

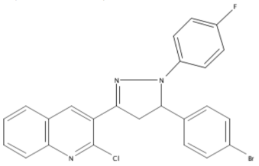

Resulting in 0 candidate

Create Keep Me Posted Alert

Contact Us | Legal

Copyright © 2020 American Chemical Society. All Rights Reserved. | 京ICP备13047075号-3

SPS - Weekly Acti....docx Removed

Show all

Type here to search

ENG IN 4:48 PM 9/10/2020

Mail - Narender Yadav - Outlook × SciFinder - Substance Answer Set × +

origin-scifinder.cas.org/scifinder/view/scifinder/scifinderExplore.jsf

Apps Gmail YouTube Maps https://origin-scifin...

CAS Solutions

SCIFINDER A CAS SOLUTION

Preferences | SciFinder Help Sign Out

Welcome Narender Yadav

Explore Saved Searches SciPlanner

Explore Substances resulted in 0 substances Return

Chemical Structure exact > **substances (0)**

Explore substances by structure: substances *exact*

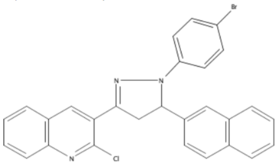

Resulting in 0 candidate

Create Keep Me Posted Alert

Contact Us | Legal

Copyright © 2020 American Chemical Society. All Rights Reserved. | 京ICP备13047075号-3

SPS - Weekly Acti....docx Removed

Show all

Type here to search

ENG IN 4:48 PM 9/10/2020
